# First-in-human phase 1 study of novel dUTPase inhibitor TAS-114 in combination with S-1 in Japanese patients with advanced solid tumors

**DOI:** 10.1007/s10637-018-0697-3

**Published:** 2018-12-04

**Authors:** Toshihiko Doi, Kiyotaka Yoh, Kohei Shitara, Hideaki Takahashi, Makoto Ueno, Satoshi Kobayashi, Manabu Morimoto, Takuji Okusaka, Hideki Ueno, Chigusa Morizane, Naohiro Okano, Fumio Nagashima, Junji Furuse

**Affiliations:** 1 Department of Experimental Therapeutics, National Cancer Center Hospital East, 6-5-1 Kashiwanoha, Kashiwa, Chiba 277-8577 Japan; 2 Department of Gastroenterology and Gastrointestinal Oncology, National Cancer Center Hospital East, 6-5-1 Kashiwanoha, Kashiwa, Chiba 277-8577 Japan; 3 Department of Gastroenterology, Kanagawa Cancer Center, 2-3-2 Nakao, Asahi-ku, Yokohama, Kanagawa 241-8515 Japan; 4 Department of Hepatobiliary and Pancreatic Oncology, National Cancer Center Hospital, 5-1-1 Tsukiji, Chuo-ku, Tokyo, 104-0045 Japan; 5 Department of Medical Oncology, Kyorin University Faculty of Medicine, 6-20-2 Shinkawa, Mitaka, Tokyo, 181-8611 Japan

**Keywords:** TAS-114, S-1, dUTPase, Solid tumor, Phase 1, Dose-escalation

## Abstract

*Background* This first-in-human phase 1 study assessed the safety of TAS-114, a novel deoxyuridine triphosphatase inhibitor, combined with S-1 to determine its maximum tolerated dose (MTD) and recommended dose (RD). *Methods* In this dose-escalation study with a 3 + 3 design, TAS-114 and S-1 were concurrently administered orally under fasting conditions at 5–240 mg/m^2^ and 30–36 mg/m^2^, respectively, in patients with advanced solid tumors. Safety, efficacy, and pharmacokinetics (PK) were evaluated. *Results* Seventy-six patients were enrolled. The MTD and RD were TAS-114 200 mg/m^2^ plus S-1 36 mg/m^2^ and TAS-114 240 mg/m^2^ plus S-1 30 mg/m^2^, respectively. Common treatment-related adverse events were anemia, lymphocytopenia, leukopenia, neutropenia, decreased appetite, rash, nausea, and pigmentation disorder. Partial response (PR) was observed in 10 patients (non-small cell lung cancer [NSCLC], *n* = 5; pancreatic neuroendocrine tumor, *n* = 2; gastric cancer, *n* = 2; gallbladder cancer, *n* = 1). Of these, four patients achieved PR despite prior treatment history with S-1. Patients administered TAS-114 exhibited linear PK and CYP3A4 induction, with no effect on the PK of S-1. *Conclusion* TAS-114 plus S-1 showed tolerable, safe, and potentially effective results. To confirm safety and efficacy, two phase 2 studies are ongoing in NSCLC and gastric cancer patients. *Clinical trial registration* ClinicalTrials.gov (NCT01610479) .

**Electronic supplementary material:**

The online version of this article (10.1007/s10637-018-0697-3) contains supplementary material, which is available to authorized users.

## Introduction

Given the complexity and variability of the molecular processes that lead to cancer development [1, 2], current cancer therapy is multifaceted. It includes molecular targeted therapy [1], immune therapy [2, 3], and other specific agents targeting cellular regulatory factors, oncogenic signalling pathways, tumor-associated angiogenesis, and host immune responses [1–3]. Despite these advances, cytotoxic agents still play an important role in systemic chemotherapy for several tumor types [4–6].

Currently, fluoropyrimidines are the cornerstone therapy for different types of cancer, and various efforts to improve their efficacy have been attempted in the past three decades. While combined treatment with chemical modulators such as leucovorin enhanced the clinical efficacy for various tumors [7], this was limited by intrinsic and acquired resistance [8]. Hence, there is a need for the development of a new, highly efficacious combination to maximize the usefulness of fluoropyrimidine therapy.

Deoxyuridine triphosphatase (dUTPase), a gatekeeper protein, is an important mediator of the antitumor efficacy of thymidylate synthase (TS)-targeted agents, such as 5-fluorouracil (5-FU) [9]. Inhibition of TS by 5-FU leads to depletion of deoxythymidine triphosphate and elevates deoxyuridine triphosphate (dUTP) and fluorodeoxyuridine triphosphate (FdUTP), which are recognized as substrates by DNA polymerases. Increased dUTP and FdUTP are misincorporated into DNA instead of deoxythymidine triphosphate. This causes DNA dysfunction, and it is one of the mechanisms of antitumor activity of 5-FU. dUTPase catalyzes the conversion of dUTP and FdUTP to deoxyuridine monophosphate and fluorodeoxyuridine monophosphate and tightly restricts 5-FU and uracil misincorporation into DNA. Suppression of dUTPase by siRNA sensitizes cancer cells to TS-inhibitors [10] and higher expression of dUTPase in tumors has been associated with resistance to 5-FU-based chemotherapy [11]. Therefore, dUTPase inhibition aimed at modulating the DNA misincorporation pathway is an attractive drug target to enhance the antitumor activity of 5-FU, and it represents a novel strategy in 5-FU-based chemotherapy.

We have developed TAS-114, a novel oral dUTPase inhibitor. While TAS-114 does not have antitumor activity in itself, it may potentially be used as part of a new treatment strategy involving 5-FU-based combination chemotherapy to enhance the antitumor activity of 5-FU through misincorporation of FdUTP and dUTP into DNA [12–14]. The mechanism of action of TAS-114 combined with 5-FU is provided in Supplementary Fig. 1. In preclinical studies, when TAS-114 was combined with 5-FU agents such as S-1 or capecitabine, it enhanced antitumor activity compared with 5-FU alone in various human cancer xenograft models [15, 16]. Previously, the first-in-human phase 1 trial of TAS-114 was conducted in healthy male volunteers to assess its pharmacokinetics (PK) and safety [14]. TAS-114 had linear PK and a favorable safety profile with single or multiple dosing.

Here, we aimed to assess the safety of TAS-114 in combination with S-1, determine its maximum tolerated dose (MTD) and recommended dose (RD), and evaluate its PK, biomarker profiles, and preliminary efficacy in patients with advanced solid tumors.

## Methods

### Study design and treatment

This was a multicenter, open-label, single-arm, dose-escalation, phase 1 study with a 3 + 3 design. The study was conducted in two parts: a dose escalation phase (Part 1) and an expansion phase (Part 2). This study was conducted in accordance with Declaration of Helsinki and Good Clinical Practice guidelines, and it was approved by the institutional review boards of the participating centers. All patients provided written informed consent.

TAS-114 and S-1 were concurrently administered orally twice daily under fasting conditions for 14 consecutive days followed by a 7-day rest, comprising a 21-day treatment cycle until disease progression, unacceptable toxicity, or withdrawal of consent occurred. In Part 1, the initial doses of TAS-114 and S-1 were 5 mg/m^2^ and 30 mg/m^2^, respectively. The dose of TAS-114 was set according to pre-clinical studies, which suggested the highest non-severely toxic dose. Dose-limiting toxicities (DLTs) at each dose level were assessed before moving on to the next dose level. The dose of TAS-114 was increased from 5 mg/m^2^ to a maximum of 240 mg/m^2^. Escalation of 100% of the TAS-114 dose was permitted until a dose of 40 mg/m^2^ was reached, after which dose increments were allowed in a tapered manner. In Japanese clinical practice, S-1 is administered at approximately 36 mg/m^2^ under fed conditions. In the present study, S-1 30 mg/m^2^ was administered under fasting conditions in accordance with standard care in Europe. The dose of S-1 could be increased to a maximum of 36 mg/m^2^ once the safety of the TAS-114 240 mg/m^2^ dose in the S-1 30 mg/m^2^ cohort was confirmed. When the dose of S-1 was to be increased, the dose of TAS-114 would be one level lower than the dose that could be safely administered in combination with 30 mg/m^2^ of S-1. The MTD was defined as the dose below the dose level at which two patients (in a cohort of up to six patients) experienced a DLT in Cycle 1. In the MTD level cohort, six patients were required to evaluate safety. DLTs were defined as any of the following treatment-related adverse events (TRAEs) occurring during Cycle 1: ≥grade 3 non-hematologic toxicity, ≥grade 3 nausea/vomiting/diarrhea lasting >48 h and uncontrolled by aggressive medications, grade 4 neutropenia lasting >7 days, febrile neutropenia, grade 4 thrombocytopenia or grade 3 thrombocytopenia associated with bleeding and requiring blood transfusion, any TRAE that prevented completion of Cycle 1 (< 80% of planned cumulative dose), or any TRAE resulting in a > 14-day delay in the initiation of Cycle 2. In Part 2, we used the dose levels determined in Part 1, and multiple doses could be selected to evaluate the safety and efficacy of specific levels.

In patients who had adverse events (AEs), a total of two dose reductions were permitted. No dose escalation was permitted once a dose reduction occurred. Dose reduction and interruption had to be implemented simultaneously for TAS-114 and S-1. In case the subsequent cycle was delayed for more than 3 weeks, study treatment was discontinued. Additional details are provided in the Supplementary Information.

### Patient population

Patients aged ≥20 years with histologically or cytologically confirmed solid tumors in whom the standard or other standard-equivalent therapy was ineffective or inappropriate were eligible for the study. In Part 2, patients for whom 5-FU chemotherapy was indicated were also eligible based on discussions between the investigator and sponsor. Other inclusion criteria included a life expectancy of ≥3 months, Eastern Cooperative Oncology Group performance status of 0 or 1, ability to receive medications orally, measurable or non-measurable lesions based on the Response Evaluation Criteria In Solid Tumors (RECIST version 1.1, 2009), and adequate hematologic, hepatic, and renal functions. Patient eligibility was confirmed by laboratory investigations within the 7 days before enrollment, which included aspartate aminotransferase or alanine aminotransferase concentrations, total bilirubin, absolute neutrophil count, platelet count, hemoglobin concentration, and creatinine clearance. Patients were excluded from the study if they had a severe disease or clinical condition (e.g., brain metastasis, active infection, heart or gastrointestinal diseases, or HIV- or hepatitis C virus-positive status). Surgery, extended-field radiotherapy, chemotherapy within 28 days (35 days for mitomycin C), local radiotherapy within 14 days, or other investigational products within 30 days prior to enrollment were not permitted. Patients who were receiving treatment known to have interactions with S-1 were excluded.

### Safety assessment

Safety assessments were done at screening and then on Days 1, 8, and 15 during the first 6 cycles; Days 1 and 15 for any subsequent cycles; at end-of-treatment; and at follow-up visits (30 days after the last administration). In these assessments, patients were monitored for hematology, serum chemistry, urinalysis, vital signs and body weight, and Eastern Cooperative Oncology Group performance status. AEs were reported at each visit using the verbatim term and coded according to the Medical Dictionary for Regulatory Activities terminology *(MedDRA®; MedDRA® trademark is registered by IFPMA on behalf of ICH)*. The severity of toxicity was graded using the *National Cancer Institute* Common Terminology Criteria for Adverse Events (Version 4.03).

### Efficacy assessment

Tumor evaluation was performed by the investigator according to RECIST 1.1. Computed tomography scanning or magnetic resonance imaging was performed at baseline and every 42 days from Day 1 of Cycle 1.

Overall response rate (ORR) was the percentage of patients in whom the best overall response was a complete response (CR) or partial response (PR) in the analysis set. CR and PR were assessed 4 weeks after the initial response for confirmation. Progression-free survival (PFS) was the period from the study enrollment day to the day of progressive disease (PD) documentation or death by any cause. If post-treatment was initiated before PD documentation, the period for PFS was censored on the last day in which progression was not observed.

### PK assessment

To evaluate TAS-114 and S-1 PK parameters, blood samples were collected on Day 1 in Cycle 1, before and 1, 2, 4, 6, 8, and 12 h after study drug administration. After the protocol amendment, blood sampling was added on Days 7 and 14 in Cycle 1 and Day 1 in Cycle 2, before and 1, 2, 4, 6, 8, and 12 h after study drug administration to evaluate the effect of CYP3A4 induction on TAS-114 PK. Similarly, to measure urine concentrations of cortisol and 6β-hydroxycortisol (6-OHF) as the indexes of CYP3A4 activity induction, 12-h urine collections were conducted on Day 0 (before the start of study drug administration), and after administration in the morning on Days 1, 7, 14, and 21 in Cycle 1. The concentrations of TAS-114, components of S-1 (tegafur [FT], 5-chloro-2,4-dihydroxypyridine [CDHP], and potassium oxonate [Oxo]), and 5-FU (a metabolite of tegafur) were measured in plasma by validated bioanalytical methods using liquid chromatography-tandem mass spectrometry at Shin Nippon Biomedical Laboratories, Ltd., Pharmacokinetics and Bioanalysis Center (Tokyo, Japan). The concentrations of cortisol and 6-OHF were measured in urine by validated bioanalytical methods using liquid chromatography-tandem mass spectrometry at Sumika Chemical Analysis Service, Ltd. (Osaka, Japan).

### Biomarker assessment

Archival tissue samples were obtained from all enrolled patients who had consented for pharmacogenomics assessment and formalin-fixed, paraffin-embedded (FFPE) specimens were created. Protein expression levels of dUTPase, TS, dihydropyrimidine dehydrogenase (DPD), thymidine phosphorylase (TP), and breast cancer 1, early onset (BRCA1) in FFPE specimens, were determined by immunohistochemistry by SRL (Tokyo, Japan). Gene expression levels of dUTPase, TS, DPD, TP, uracil-DNA glycosylase, apurinic/apyrimidinic endodeoxyribonuclease 1, DNA polymerase beta, BRCA1, and breast cancer 2 in FFPE specimens, early onset, were determined by Gentris Corporation (Morrisville, NC, USA) using quantitative reverse transcription polymerase chain reaction. Assessment method details are provided in the Supplementary Information*.*

### Statistical analysis

No formal sample size calculations were performed. To obtain preliminary information on the MTD and safety, we planned a maximum enrollment of 80 patients for DLT evaluations (Part 1) and a maximum enrollment of 40 patients for the full analysis set (FAS) (Part 2). DLTs were evaluated based on the patients in Part 1 who experienced DLTs and those who did not experience DLTs but received ≥80% of the planned total dose of the study drug in Cycle 1. Safety data were analyzed in patients who received at least one dose of TAS-114 and S-1. The efficacy analysis was based on the FAS, defined as all patients who received study treatment in Part 1 or Part 2, those who met all inclusion criteria and none of the exclusion criteria and provided at least one measured value for efficacy endpoints after the start of study drug administration.

The incidences of AEs and grade ≥3 AEs and the number of patients with AEs were calculated by event and severity. ORR and PFS were analyzed in the FAS; 95% confidence intervals (CIs) were estimated. Summary statistics were presented by dose level. The estimation of plasma PK parameters was performed using Phoenix WinNonlin Professional (Version 6.1 or later; Pharsight Corporation, Sunnyvale, CA, USA) according to the non-compartmental method.

## Results

### Patients

A total of 76 patients were enrolled at four sites in Japan between May 2012 and April 2016, of whom all patients (Part 1, *n* = 48; Part 2, *n* = 28) were eligible and received at least 1 dose of TAS-114 and S-1; 64 and 12 patients were assigned to the S-1 30 mg/m^2^ and S-1 36 mg/m^2^ cohorts, respectively. Baseline characteristics are listed in Table 1. The most frequently reported cancer types were pancreatic cancer (*n* = 17), non-small cell lung cancer (NSCLC) (*n* = 16), and colorectal cancer (*n* = 10). The majority of patients (74 patients) had received at least one prior chemotherapy for metastatic disease, of whom 42 patients were treated with a 5-FU containing regimen; two patients had not received any prior chemotherapy for metastatic disease.

**Table 1:** Baseline patient characteristics

	S-1 30 mg/m^2^ (*n* = 64)	S-1 36 mg/m^2^ (*n* = 12)	Total (*n* = 76)
	*n*	*n*	*n*
Age, years			
Median (range)	64.0 (34–81)	66.0 (47–73)	64.0 (34–81)
Sex			
Male	41	10	51
Female	23	2	25
ECOG PS			
0	34	6	40
1	30	6	36
Cancer type			
Pancreas	17	0	17
NSCLC	14	2	16
Colorectal	9	1	10
Bladder	4	3	7
p-NET	4	0	4
Gastric	3	0	3
Other	13	6	19
Number of prior therapies			
1	7	2	9
2	19	3	22
3	20	4	24
4	8	1	9
≥5	8	2	10
Number of prior 5-FU therapies	38	4	42

Abbreviations: *5-FU* 5-fluorouracil, *ECOG PS* Eastern Cooperative Oncology Group performance status, *NSCLC* non-small cell lung cancer, *p-NET* pancreatic neuroendocrine tumor

**Fig. 1:**
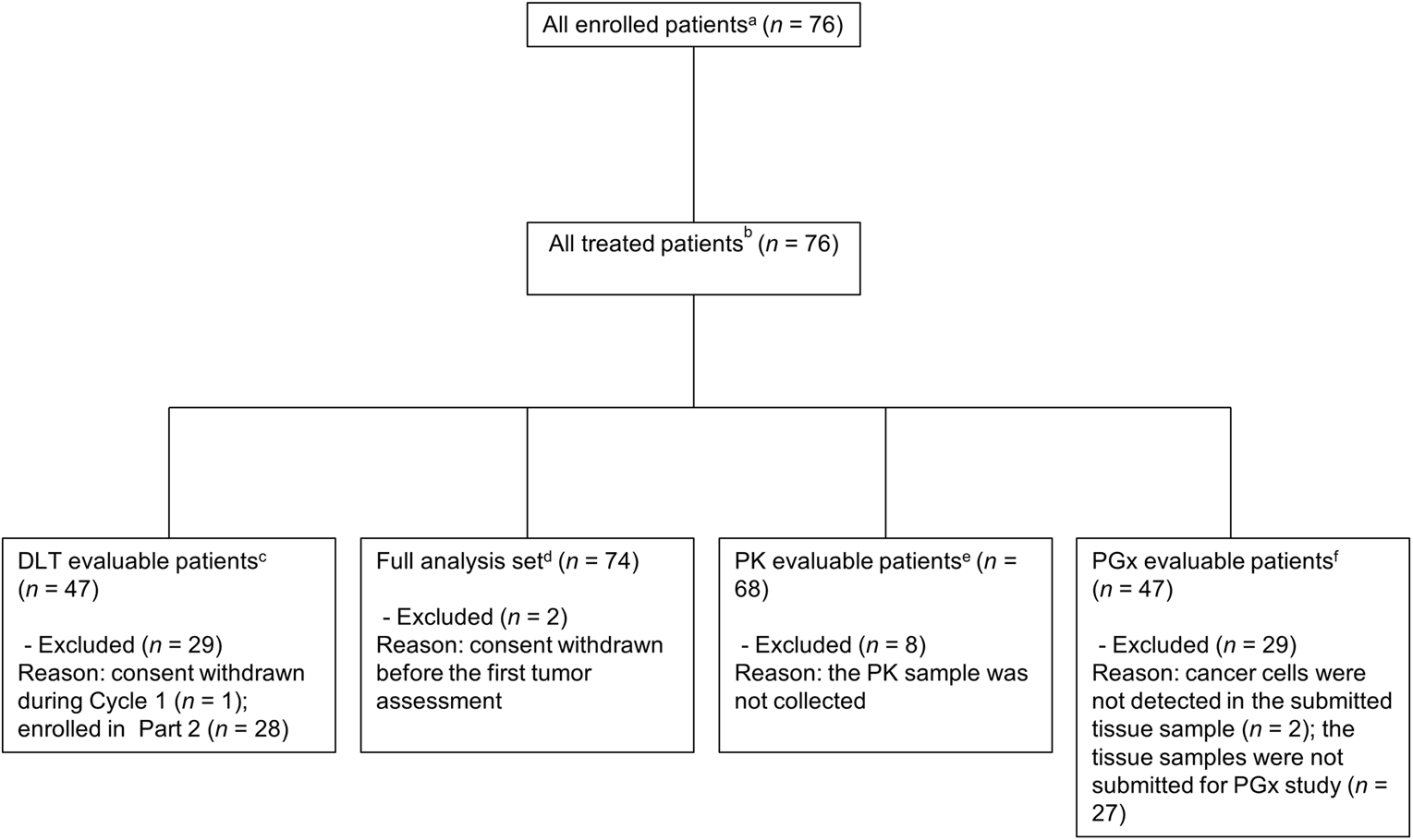
Patient analysis sets. *Abbreviations: DLT,* dose-limiting toxicity; *PGx,* pharmacogenomics; *PK,* pharmacokinetic. ^a^ All patients enrolled in Part 1 or Part 2. ^b^ Of patients enrolled in Part 1 or Part 2, all treated patients including those who received at least 1 dose of TAS-114 and S-1. ^c^ DLTs were evaluated based on the patients in Part 1 who experienced DLTs and those who did not experience DLTs but received ≥80% of the planned total dose of the study drug in Cycle 1. ^d^ Of patients who received study treatment in Part 1 or Part 2, the full analysis set included those who met all inclusion criteria, did not meet any of the exclusion criteria, and provided at least one measured value for efficacy endpoints after the start of study drug administration. ^e^ PK evaluable patients included those who received TAS-114 and S-1 at the assigned doses and provided blood and urine samples needed for calculation of PK parameters. ^f^ Of patients who received study treatment, PGx evaluable patients included those who provided measured values for PGx analyses

Patient analysis sets are summarized in Fig. 1. Of the 48 patients in Part 1, one patient, who discontinued study treatment in Cycle 1 due to withdrawal of consent, was excluded from the DLT evaluation. The FAS comprised 74 patients because two patients discontinued study treatment due to withdrawal of consent before the first tumor assessment.

### Dose escalation, DLTs, MTD, and RD

A summary of dose escalation and DLTs is shown in Table 2. Forty-seven patients in Part 1 were evaluable for DLTs. At an initial S-1 dose of 30 mg/m^2^, DLTs were reported in one of six patients in the TAS-114 10 mg/m^2^ cohort (grade 2 platelet count decreased with >14-day delay in Cycle 2 initiation), and one of six patients in the TAS-114 240 mg/m^2^ cohort (grade 3 aspartate aminotransferase increased). As the safety of the TAS-114 240 mg/m^2^ dose in the S-1 30 mg/m^2^ cohort was confirmed, the dose of S-1 was escalated to 36 mg/m^2^. The starting dose of TAS-114 in the S-1 36 mg/m^2^ cohort was set at 200 mg/m^2^ according to the protocol. As no DLTs were reported in the three initially enrolled patients, the TAS-114 dose was escalated to 240 mg/m^2^. Because two of five patients reported DLTs (grade 4 platelet count decreased and grade 3 dermatitis bullous), the lower dose level cohort (200 mg/m^2^) was expanded to six patients. As no other patients developed DLTs, the MTD was determined to be TAS-114 200 mg/m^2^ plus S-1 36 mg/m^2^. However, from a safety and treatment continuity perspective, the RD was determined to be TAS-114 240 mg/m^2^ plus S-1 30 mg/m^2^ because all patients who received the MTD required dose reduction or dose interruption within Cycle 3.

**Table 2:** Summary of dose escalation and DLTs (DLT evaluable patients, *n* = 47)

S-1 (mg/m^2^)	TAS-114 (mg/m^2^)	Part 1 patients treated, *n*	Patients evaluable for DLTs, *n*	Patients with DLTs, *n*	Description of DLTs
30	5	3	3	0	–
	10	6	6	1	Platelet count decreased (G2) with a > 14-day delay in Cycle 2 initiation
	20	3	3	0	–
	40	3	3	0	–
	60	3	3	0	–
	90	3	3	0	–
	120	3	3	0	–
	160	3	3	0	–
	200	3	3	0	–
	240	6	6	1	Aspartate aminotransferase increased (G3)
36	200	7	6	0	–
	240	5	5	2	Platelet count decreased (G4)
					Dermatitis bullous (G3)

Abbreviation: *DLT* dose-limiting toxicities

### Treatment exposure, dose modification, and discontinuation

The median relative dose intensity (RDI) of TAS-114 and S-1 for all patients was 86% and 87%, respectively. When summarized by dose level, the RDI of the RD (TAS-114 240 mg/m^2^ plus S-1 30 mg/m^2^) cohort was 91% for both TAS-114 and S-1, and the RDI of the MTD (TAS-114 200 mg/m^2^ plus S-1 36 mg/m^2^) cohort was 63% for TAS-114 and 68% for S-1.

Among all patients, 24% and 26% of patients had at least one dose reduction of TAS-114 and S-1, respectively. Dose interruption and dosing delay in a subsequent cycle was required in 60% and 46% of patients, respectively. The major reasons for TRAEs leading to dose reduction, dose interruption, and dosing delay in a subsequent cycle were rash, fatigue, nausea, and thrombocytopenia. In terms of the dose level, in the RD cohort, 30% of patients had a dose reduction, 50% of patients had a dose interruption, and 40% of patients had a dosing delay; in the MTD cohort, all patients had both a dose reduction and interruption, and 80% of patients had a dosing delay.

Reasons for discontinuation of the study treatment included disease progression as defined by RECIST criteria (56 patients), clinical progression (9 patients), toxicity (7 patients; neutrophil count decreased, *n* = 2; platelet count decreased, *n* = 1; deep vein thrombosis, *n* = 1; pneumonitis, *n* = 1; interstitial lung disease, *n* = 1; and dermatitis bullous *n* = 1) and withdrawal of consent (4 patients).

### Safety

TRAEs occurring in ≥20% of patients are summarized in Table 3. Seventy-one of 76 patients (93.4%) experienced at least one TRAE during all cycles. Overall, most of the TRAEs were grade ≤2 and clinically manageable. The incidences of TRAEs in the S-1 36 mg/m^2^ cohort were higher than those in the S-1 30 mg/m^2^ cohort. The most frequently reported TRAEs (≥30% of patients) were anemia (63%), lymphocytopenia (41%), leukopenia (41%), decreased appetite (34%), rash (33%), and nausea (31%) in the S-1 30 mg/m^2^ cohort, and decreased appetite (83%), rash (67%), anemia (67%), nausea (67%), fatigue (67%), thrombocytopenia (58%), lymphocytopenia (50%), neutropenia (50%), leukopenia (50%), stomatitis (42%), and pigmentation disorder (42%) in the S-1 36 mg/m^2^ cohort. Common grade ≥3 TRAEs were lymphocytopenia (33%) and anemia (27%) in the S-1 30 mg/m^2^ cohort, and lymphocytopenia (50%), anemia (42%), leukopenia (42%), thrombocytopenia (42%), and neutropenia (33%) in the S-1 36 mg/m^2^ cohort.

**Table 3:** Summary of treatment-related adverse events occurring in ≥20% of patients in the S-1 30 mg/m^2^ and 36 mg/m^2^ cohorts (all treated patients, *n* = 76)

S-1 (mg/m^2^)	30																						36					
TAS-114 (mg/m^2^)	5 (*n* = 3)		10 (*n* = 6)		20 (*n* = 3)		40 (*n* = 3)		60 (*n* = 3)		90 (*n* = 3)		120 (*n* = 10)		160 (*n* = 20)		200 (*n* = 3)		240 (*n* = 10)		Total (*n* = 64)		200 (*n* = 7)		240 (*n* = 5)		Total (*n* = 12)	
Grades	All	≥3	All	≥3	All	≥3	All	≥3	All	≥3	All	≥3	All	≥3	All	≥3	All	≥3	All	≥3	All	≥3	All	≥3	All	≥3	All	≥3
Anemia	–	–	–	–	3 (100)	1 (33)	1 (33)	1 (33)	1 (33)	–	3 (100)	2 (67)	6 (60)	5 (50)	16 (80)	4 (20)	3 (100)	1 (33)	7 (70)	3 (30)	40 (63)	17 (27)	4 (57)	2 (29)	4 (80)	3 (60)	8 (67)	5 (42)
Diarrhea	–	–	–	–	–	–	1 (33)	–	1 (33)	–	1 (33)	–	2 (20)	1 (10)	7 (35)	–	–	–	3 (30)	–	15 (23)	1 (2)	–	–	–	–	–	–
Nausea	–	–	1 (17)	–	1 (33)	–	–	–	2 (67)	–	2 (67)	–	2 (20)	–	9 (45)	–	–	–	3 (30)	–	20 (31)	–	7 (100)	1 (14)	1 (20)	–	8 (67)	1 (8)
Stomatitis	–	–	–	–	–	–	–	–	1 (33)	–	–	–	2 (20)	–	4 (20)	–	1 (33)	–	–	–	8 (13)	–	3 (43)	1 (14)	2 (40)	–	5 (42)	1 (8)
Fatigue	–	–	1 (17)	–	–	–	1 (33)	–	1 (33)	–	–	–	2 (20)	–	6 (30)	1 (5)	2 (67)	1 (33)	5 (50)	–	18 (28)	2 (3)	5 (71)	–	3 (60)	–	8 (67)	–
Lymphocytopenia	–	–	1 (17)	–	–	–	3 (100)	2 (67)	–	–	2 (67)	2 (67)	5 (50)	4 (40)	9 (45)	7 (35)	1 (33)	1 (33)	5 (50)	5 (50)	26 (41)	21 (33)	3 (43)	3 (43)	3 (60)	3 (60)	6 (50)	6 (50)
Neutropenia	1 (33)	–	1 (17)	–	1 (33)	–	3 (100)	–	1 (33)	–	–	–	4 (40)	1 (10)	5 (25)	3 (15)	1 (33)	–	2 (20)	–	19 (30)	4 (6)	3 (43)	1 (14)	3 (60)	3 (60)	6 (50)	4 (33)
Thrombocytopenia	–	–	2 (33)	–	1 (33)	–	–	–	1 (33)	–	–	–	2 (20)	–	2 (10)	–	–	–	1 (10)	1 (10)	9 (14)	1 (2)	4 (57)	2 (29)	3 (60)	3 (60)	7 (58)	5 (42)
Leukopenia	–	–	3 (50)	–	2 (67)	–	3 (100)	1 (33)	1 (33)	–	2 (67)	–	4 (40)	2 (20)	7 (35)	3 (15)	1 (33)	–	3 (30)	–	26 (41)	6 (9)	3 (43)	2 (29)	3 (60)	3 (60)	6 (50)	5 (42)
Decreased appetite	–	–	1 (17)	–	1 (33)	–	–	–	1 (33)	–	1 (33)	–	5 (50)	1 (10)	7 (35)	–	1 (33)	–	5 (50)	–	22 (34)	1 (2)	7 (100)	–	3 (60)	–	10 (83)	–
Rash	1 (33)	–	–	–	–	–	1 (33)	–	1 (33)	–	–	–	4 (40)	–	8 (40)	–	–	–	6 (60)	–	21 (33)	–	5 (71)	–	3 (60)	–	8 (67)	–
Pigmentation disorder	1 (33)	–	1 (17)	–	–	–	2 (67)	–	–	–	–	–	1 (10)	–	9 (45)	–	1 (33)	–	4 (40)	–	19 (30)	–	3 (43)	–	2 (40)	–	5 (42)	–

Data are *n* (%)

Twenty-five patients (32.9%) experienced at least one serious AE (SAE) during all cycles. The most frequently reported SAEs, which occurred in two or more patients, were decreased appetite (*n* = 4), abdominal pain (*n* = 3), malaise (*n* = 2), pyrexia (*n* = 2) in the S-1 30 mg/m^2^ cohort, and pyrexia (*n* = 2) in the S-1 36 mg/m^2^ cohort. No unexpected SAEs or treatment-related deaths occurred in this study.

### Efficacy

**Fig. 2:**
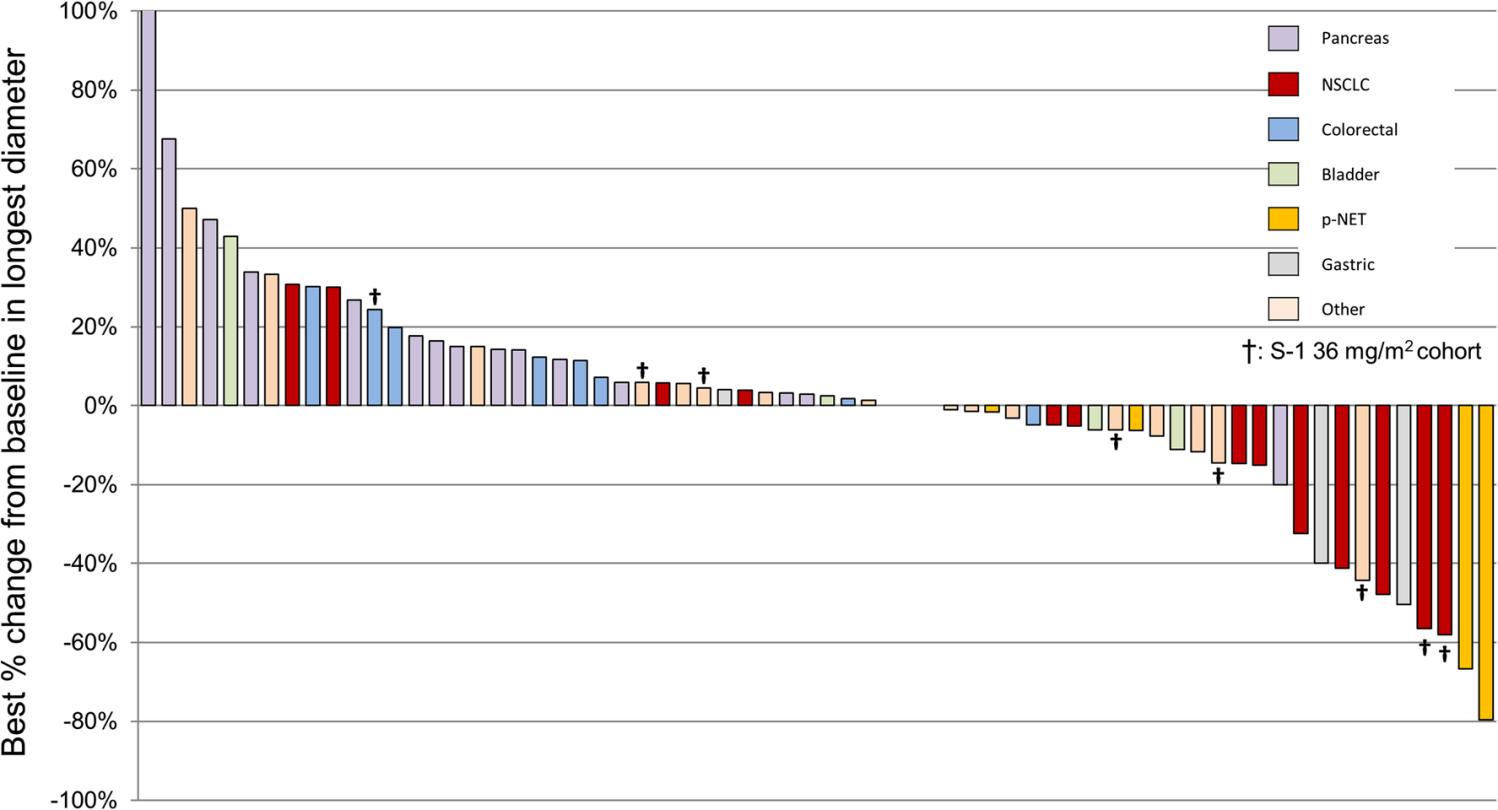
Waterfall plots showing the best percent change from baseline in the longest diameter of the target lesions for S-1 30 mg/m^2^ and S-1 36 mg/m^2^. *Abbreviations: NSCLC,* non-small cell lung cancer; *p-NET,* pancreatic neuroendocrine tumor. Analysis Set: Full Analysis Set. Dose of S-1: Level 3 = 30 mg/m^2^ and 36 mg/m^2^. Change from baseline (%) = min ([value at each date - baseline value] / baseline value × 100) ^†^ S-1 36 mg/m^2^

Among the 74 evaluable patients in the FAS, PR was observed in 10 patients (NSCLC, *n* = 5; pancreatic neuroendocrine tumor [p-NET], *n* = 2; gastric cancer, *n* = 2; and gallbladder cancer, *n* = 1). Of the 10 patients, PR was confirmed in seven patients (NSCLC, *n* = 3; p-NET, *n* = 2; gastric cancer, *n* = 1; and gallbladder cancer, *n* = 1), resulting in an ORR of 9.5% (95% CI, 3.9–18.5). Furthermore, four patients (NSCLC, *n* = 1; gastric cancer, *n* = 2; and gallbladder cancer, *n* = 1) achieved PR despite prior treatment history with S-1. Best response of stable disease (SD) was observed in 28 patients (37.8%). Figure 2 shows the maximum shrinkage in the diameter of the target lesions from baseline.

The ORR, PFS, and number of prior systemic regimens by cancer type are shown in Supplementary Table 1. Clinical activity was observed for NSCLC (ORR and median PFS, 18.8% and 4.1 months, respectively), p-NET (ORR and PFS, 50% and 14 months, respectively), and gastric cancer (ORR and PFS, 33.3% and 2.8 months, respectively).

### PK

**Fig. 3:**
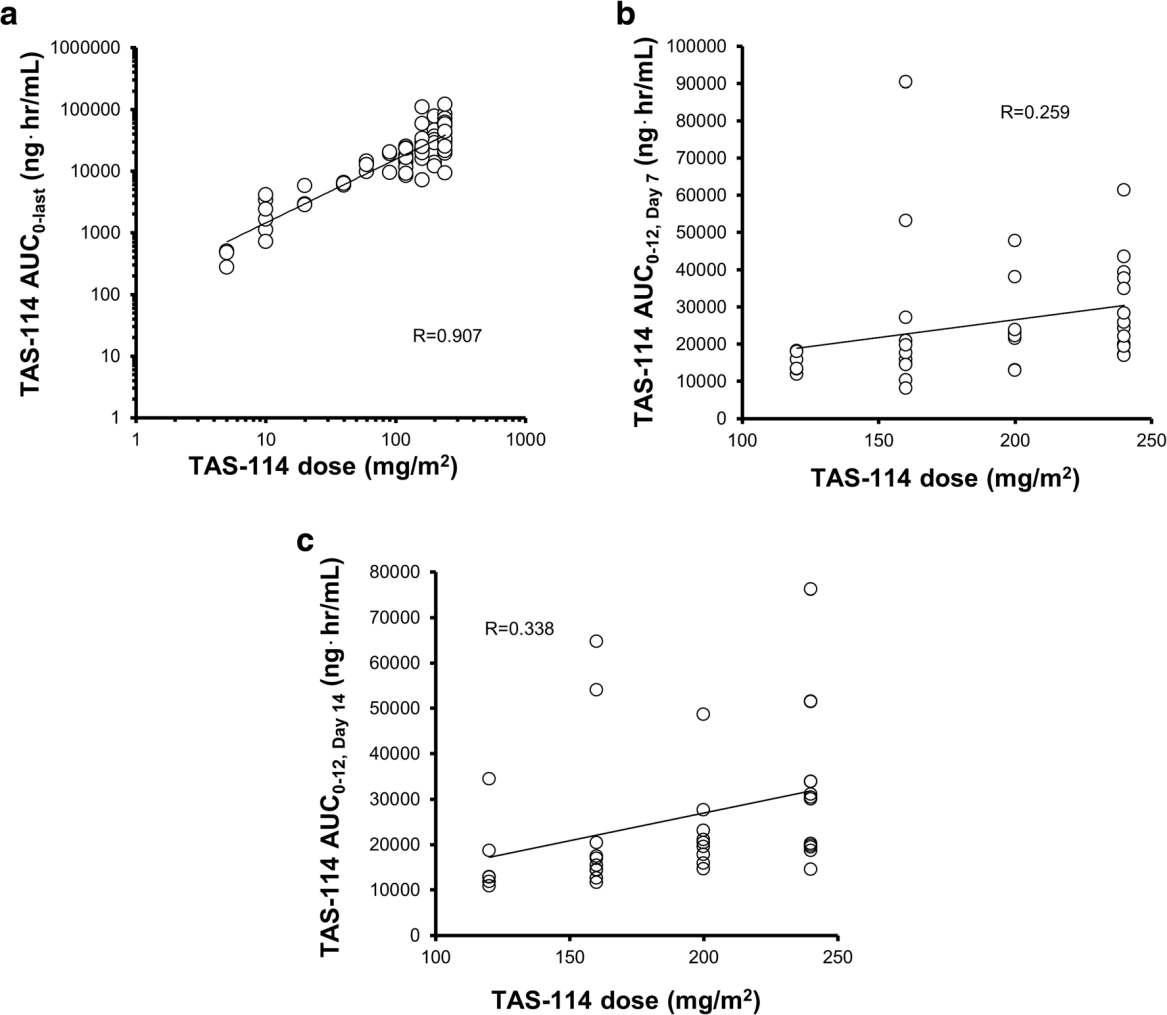
Pharmacokinetics. a. Correlation between TAS-114 AUC_0-last_ on C1D1 and BSA dose of TAS-114. b. Correlation between TAS-114 AUC_0–12_ on C1D7 and BSA dose of TAS-114. c. Correlation between TAS-114 AUC_0–12_ on C1D14 and BSA dose of TAS-114. *Abbreviations: AUC*_*0-last*_*,* area under plasma concentration-time curve from time 0 to last quantifiable concentration; *AUC*_*0–12*_*,* area under the plasma concentration-time curve from time 0 to 12 h; *BSA,* body surface area; *C1D1,* Day 1 of Cycle 1; *C1D7,* Day 7 of Cycle 1; *C1D14,* Day 14 of Cycle 1

PK parameters of TAS-114 and S-1 components on Days 1, 7, and 14 in Cycle 1 are shown in Supplementary Table 2A and 2B. The area under the curve (AUC) of TAS-114 after single administration increased in a dose-proportional manner up to 240 mg/m^2^ with large inter-individual variations (correlation coefficient [R] of 0.907), but repeated administrations of TAS-114 diminished the correlations between the TAS-114 dose and AUC (Day 7 and Day 14 in Cycle 1: R 0.259 and 0.338, respectively) (Fig. 3a–c). The administration of TAS-114 did not affect the PK of S-1 components after S-1 administration (Supplementary Fig. 2a, 2b, 2c, and 2d). The AUC for 5-FU after the administration of S-1 30 mg/m^2^ under fasting conditions was comparable to that after S-1 36 mg/m^2^ under fed conditions [17], which is the approved dose in Asian countries (Supplementary Fig. 3a and 3b).

The comparison of the ratio of urine 6-OHF concentration to urine cortisol concentration (6-OHF/F) on Day 0 with that on Days 1, 7, 14, and 21 in Cycle 1 at a TAS-114 dose of 120, 160, 200, and 240 mg/m^2^ is shown in Supplementary Fig. 4a, 4b, 4c, and 4d. Compared with that on Day 0, the 6-OHF/Fs on Days 1, 7, 14, and 21 at TAS-114 120 mg/m^2^ were not significantly different. At a TAS-114 dose of 160 mg/m^2^ and over, 6-OHF/Fs on Day 7 and 14 were significantly increased, suggesting that TAS-114 induces CYP3A4 activity at 160 mg/m^2^ and higher doses. Conversely, the values of 6-OHF/F after 1-week cessation of chemotherapy were similar to those on Day 0.

### Biomarker assessment

Archival tissue samples were collected from 49 patients, from whom 47 samples were evaluable for biomarker analysis. The relationships between mRNA or protein expression levels of nucleic acid-metabolising enzymes (dUTPase, TS, DPD, and TP) and DNA repair enzymes (uracil-DNA glycosylase, apurinic/apyrimidinic endodeoxyribonuclease 1, DNA polymerase beta, BRCA1, and breast cancer 2, early onset) in tumors, disease control, and PFS were evaluated (Supplementary Tables 3 and 4). The median nuclear BRCA1 protein expression level was significantly lower in the CR + PR + SD group (*n* = 15) versus the PD group (*n* = 29, *P* = 0.004); median nuclear dUTPase protein expression level was significantly lower in the CR + PR + SD group (*n* = 15) versus the PD group (*n* = 31, *P* = 0.021). There were no statistically significant differences in the other parameters.

Patients were then divided into low- and high-expression groups using the median protein gene expression level as the cut-off value. The median PFS in the groups with high (*n* = 23) and low (*n* = 21) BRCA1 protein expression was 1.5 months (95% CI, 1.4–2.8) and 3.9 months (95% CI, 2.5–5.6), respectively (hazard ratio, 1.90 [95% CI, 1.00–3.60], *P* = 0.045). The median PFS in the groups with high (*n* = 25) and low (*n* = 22) dUTPase protein expression in the nucleus was 2.0 months (95% CI, 1.4–2.8) and 3.9 months (95% CI, 1.5–5.8), respectively (hazard ratio, 1.92 [95% CI, 1.02–3.61], *P* = 0.038).

## Discussion

This phase 1 study is the first clinical trial to evaluate a combination therapy of TAS-114 and S-1 in patients with advanced solid tumors. In this study, the RD was determined to be TAS-114 240 mg/m^2^ plus S-1 30 mg/m^2^ because all patients who received the MTD required dose reduction or dose interruption resulting in lower RDI (63% and 68%, respectively), and the MTD was not considered to be acceptable in a clinical setting.

Although TAS-114 has a moderate DPD inhibitory effect, the PK profile of 5-FU was unaffected by TAS-114 as S-1 already contains a strong DPD inhibitor, CDHP. The PK profile of CDHP, Oxo, and FT, S-1 components, was unaffected as well. Based on in vivo efficacy results, the estimated effective dose of TAS-114 was considered to be the dose at which the TAS-114 AUC was approximately 2000–4000 ng·h/mL (data not shown). Target exposure was achieved at dose level 3 (3835 ng·hr/mL); however, we decided to continue dose escalation because clear safety and efficacy effects were not observed. A pharmacodynamics assessment to evaluate the biological inhibitory activity of TAS-114 was not conducted in this study. We considered the possibility of further escalating the TAS-114 dose above 240 mg/m^2^ in the S-1 30 mg/m^2^ cohort, but we decided against it because induction of CYP3A4 activity was observed at TAS-114 160 mg/m^2^ and over, which is a good substrate for CYP3A4. This result indicated weak correlations between the TAS-114 dose and AUC after repeated administrations. A previous study suggested that TAS-114 has no significant potential of autoinduction at 150 mg/body or below; however, our study showed induction of CYP3A4 activity at a higher dose of TAS-114 [14].

The most common TRAEs (≥30%) in both the S-1 30 mg/m^2^ and S-1 36 mg/m^2^ cohorts were anemia, lymphocytopenia, leukopenia, decreased appetite, rash, and nausea. Overall safety profiles of this combination therapy were consistent with those commonly observed in past studies of S-1 monotherapy, except for anemia and rash [18–22]. The incidence of these events seemed to be higher than that in previous studies; however, these events were manageable by dose modification and symptomatic therapy. Notably, none of the patients discontinued the study due to these events. Although an analysis of the correlation between TAS-114 exposure and incidence of anemia and rash was not performed, these events may be useful pharmacodynamics markers.

This study included patients heavily pre-treated with several TAS-114 dose levels and heterogeneous tumor types; however, PR was observed in 10 patients, of whom four patients achieved PR, regardless of treatment history with S-1. In addition, particularly promising efficacy was observed in patients with NSCLC, p-NET, and gastric cancer. For example, despite the later setting, this combination showed favorable PFS (4.1 vs. 2.86 months) and ORR (18.8% vs. 8.3%) in NSCLC compared with S-1 monotherapy in a previous phase 3 study. Given that TAS-114 did not have an effect on 5-FU exposure, dUTPase inhibition by TAS-114 may contribute to antitumor activity.

A preclinical study showed that suppression of DNA repair proteins involved in homologous recombination repair by siRNA in HeLa cells increased their sensitivity to TAS-114 combined with the 5-FU metabolite 5-fluoro-2′-deoxyuridine [23]. In this study, the expression level of BRCA1 in patients with CR, PR, or SD was lower than that in patients with PD. These results suggest that patients with low BRCA1 level may be more sensitive to TAS-114 with S-1 than those with high BRCA1 level. Further examination is required because the analysis set in this study included various tumor types and various doses of TAS-114 and S-1.

While combination therapy consisting of S-1 and other chemotherapeutic agents has been proven to be clinically effective, the efficacy of that combination could be limited given the emergence of resistance to 5-FU. Previous studies have reported that dUTPase may be one of the causal factors for resistance to 5-FU-based chemotherapies [11, 24]. In the present study, both 5-FU naïve and refractory patients showed clinical benefits when receiving S-1 combined with TAS-114. Altogether, this evidence suggests that inhibition of dUTPase may be a novel approach in enhancing the activity of 5-FU-based chemotherapy.

Our study had a small sample size and only Japanese patients were enrolled. In Caucasians and East Asians, different tolerability for fluoropyrimidines was reported despite similar PK profiles in the two populations [25, 26]. A phase 1 trial in Europe investigated the tolerability of the combination therapy in European patients with advanced solid tumors, and it suggested that the combination therapy had a tolerable safety profile consistent with that in Japanese patients [27]. Furthermore, as S-1 exerts its own anti-tumor effect [18–20, 28–31], the contribution of TAS-114 to the anti-tumor effect needs to be confirmed in further studies. Currently, two phase 2 studies are underway to evaluate the contribution of TAS-114 to the antitumor effect of S-1: a randomized, international phase 2 study of TAS-114 plus S-1 vs. S-1 monotherapy in patients with NSCLC who are 5-FU naïve (ClinicalTrials.gov Identifier: NCT02855125) and a phase 2 study evaluating the efficacy and safety of TAS-114 plus S-1 in patients with gastric cancer previously treated with 5-FU (UMIN Clinical Trials Registry Identifier: UMIN000028329).

In summary, the RD was determined to be TAS-114 240 mg/m^2^ plus S-1 30 mg/m^2^. Combined treatment with TAS-114 and S-1 was well tolerated, safe, and potentially effective for patients with advanced solid tumors.

## Electronic supplementary material

ESM 1 Supplementary information. This section includes a description of the rationale for the initial dose of TAS-114 and S-1, rationale for treatment administration between meals, additional details of dose adjustment in Part 1, and details of biomarker assessment. (DOC 1.17 mb)

## Data Availability

Data generated or analyzed during this study are on file with Taiho Pharmaceutical Co., Ltd. and are not publicly available. Inquiries for data access may be sent to the following e-mail address: TOIAWASE@taiho.co.jp.
