# Supplementary material for: First-in-human phase 1 study of novel dUTPase inhibitor TAS-114 in combination with S-1 in Japanese patients with advanced solid tumors
Source: Invest New Drugs. 2018 Dec 4;37(3):507–18. doi: 10.1007/s10637-018-0697-3 (PMC6538570; doi:10.1007/s10637-018-0697-3)
Supplement: Supplementary file 1 — Supplementary information. This section includes a description of the rationale for the initial dose of TAS-114 and S-1, rationale for treatment administration between meals, additional details of dose adjustment in Part 1, and details of biomarker assessment. (DOC 1.17 mb) [file 10637_2018_697_MOESM1_ESM.doc]

**Supplementary information.**

***Rationale for the initial dose of TAS-114***

The initial dose of TAS-114 was determined in accordance with the “Guidelines for non-clinical assessment of anti-tumor drugs” [1, 2]. A 4-week multiple oral administration toxicity study for the concomitant use of TAS-114 and S-1 was conducted in dogs, which were chosen as the experimental animal species because they have high sensitivity to S-1. In the study, the highest non-severely toxic dose of TAS-114 was 2.5 mg/kg when S-1 was co-administered at the dose equivalent to the human maximum tolerated dose (MTD). Based on the study results of a phase I study in healthy adults, one-sixth of the area under the concentration-time curve during the 24 hours after administration of TAS-114 2.5 mg/kg in dogs was considered an equivalent dose to the area under the curve in humans after TAS-114 10.2 mg/m^2^/day administration. As a result, 5 mg/m^2^ (Level 1) was considered the appropriate initial dose of TAS-114 in co-administration with S-1. In addition, eight categories were set for the dose (per dosing) of TAS-114 by body surface area (BSA) corresponding to those of S-1.

***Rationale for the initial dose of S-1***

The approved dose of S-1 monotherapy in Japan is approximately 36 mg/m^2^. The sponsor considered it appropriate that the initial dose of S-1 in combination with TAS-114 should be reduced to approximately 80% of the approved dose, 30 mg/m^2^, taking into account the risk of increased toxicity. Although the dosage (per dosing) of S-1 by BSA was divided into three categories in the package insert of S-1, the dosage categories in this study were set to match the categories used in European Union (EU) and the United States (US) in order to equalize the exposure to 5-FU after administration of S-1, regardless of body type and taking into account the results of the population pharmacokinetic (PK) analyses [3]. Eight categories were set for the dose of S-1 by BSA. Based on the reasons mentioned above, administration of S-1 was performed by combining 15- and 20-mg capsules instead of the approved combination of 20- and 25-mg capsules.

***Rationale for treatment administration between meals***

The effects of meals on plasma concentrations of TAS-114 in humans were investigated in a phase 1 single- and multiple-administration study in healthy adults. PK parameters after single administration of TAS-114 at 60 mg were compared between administrations under fasting and fed conditions. The results showed that the mean maximum concentration (C_max_) was higher when administered under fed conditions compared with fasting conditions; however, no significant difference was detected in any PK parameter. These results suggest that the mean plasma concentration of TAS-114 would not be greatly influenced by meals.

Clinical pharmacological studies [4, 5], as well as population PK analyses, have been conducted in EU and the United States to examine the effects of meals on each component of S-1 and its metabolites. The results showed that the plasma concentration of potassium oxonate (Oxo) was significantly reduced when administered after a meal. In the summaries of product characteristics for the EU and the US, administration between meals is recommended to account for the effects of gastrointestinal toxicity in Europeans and Americans. As simultaneous global development was planned for this medical treatment, and taking into account the effect of meals on the plasma concentration of Oxo, S-1 was to be administered between meals as in the EU and the US, and therefore TAS-114 was to be administered simultaneously between meals.

***Additional details of dose adjustment in Part 1***

In principle, if the dose of S-1 was reduced, the starting dose of TAS-114 was to be the MTD. If the dose of S-1 was escalated, the starting dose of TAS-114 was to be one dose level lower than the MTD or the maximum dose at which TAS-114 was safely administrable in combination with S-1 at dose level 3 (30 mg/m^2^). However, if the sponsor (in conjunction with the investigators or medical expert) judged that there was a need to further reduce the starting dose of TAS-114, based on the PK profile and administration continuity, the starting dose of TAS-114 could be changed.

***Details of biomarker assessment***

For gene expression analysis, RNA was extracted using the Paradise Plus Whole Transcript Reverse Transcription Reagent System (MDS Analytical Technologies, Sunnyvale, CA) and the SuperScript VILO cDNA Synthesis Kit (Thermo Fisher Scientific, Waltham, MA) was used to reverse transcribe mRNA into cDNA. cDNA was pre-amplified using the Taqman PreAmp Master Mix kit (Thermo Fisher Scientific), and mRNA levels were analyzed using Applied Biosystems ViiA7 Real-Time PCR System (Thermo Fisher Scientific). Beta actin (ACTB) was used as the endogenous control. The primers and probe were designed using Primer Express version 3.0 (Thermo Fisher Scientific). The protein expression levels were measured by Taiho Pharmaceutical Immunohistochemistry. The primary rabbit polyclonal antibodies for deoxyuridine triphosphatase and thymidylate synthase and the primary mouse monoclonal antibodies against dihydropyrimidine dehydrogenase and thymidine phosphorylase were prepared by Taiho Pharmaceutical Co. Ltd (Tokyo, Japan).

Regarding storage, remains of nucleic acid samples extracted from unstained slides at the measurement laboratory are stored for 10 years following completion of the study period. This is to ensure that they are available for exploratory measurement of factors expected to have an association with the clinical effects and adverse drug reactions of TAS-114, as needed in the future. Specimens stained by Gentris Corporation (Morrisville, NC, US) and SRL (Tokyo, Japan) as well as unstained specimens are stored at these companies for 10 years after the study period is completed. Prepared hematoxylin-eosin (HE)-stained specimens are stored for 10 years after the study period is completed. When the storage period expires, specimens are discarded at the sample measurement laboratory after the deletion of ID numbers under the responsibility of Taiho Pharmaceutical Co., Ltd.

**References**

1. Pharmaceuticals and Medical Devices Agency. Non-clinical evaluation of ICH-S9 antineoplastic drug <https://www.pmda.go.jp/int-activities/int-harmony/ich/0061.html> Accessed 1 June 2018

2. International Conference On Harmonisation of Technical Requirements For Registration of Pharmaceuticals for Human Use. Nonclinical Evaluation for Anticancer Pharmaceuticals. October 2009. <https://www.pmda.go.jp/files/000156321.pdf> Accessed 1 June 2018

3. Yoshida K, Ikeda K, Yoshisue K, Rodriguez W, Bodoky G, Moiseyenko V, Lichinitser M, Saito K, Benedetti FM, Ajani JA, The FLAGS Trial Investigators Group (2012) Population pharmacokinetic (PPK) analysis for 5-FU, tegafur (FT), gimeracil (CDHP), and oteracil potassium (Oxo) in the eight clinical studies of S-1 in Western patients with advanced solid tumors. J Clin Oncol 29 (Suppl 4): 53 (abstract 53)

4. Scheulen ME, Saito K, Hilger RA, Mende B, Zergebel C, Strumberg D (2012) Effect of food and a proton pump inhibitor on the pharmacokinetics of S-1 following oral administration of S-1 in patients with advanced solid tumors. Cancer Chemother Pharmacol 69: 753–761. https://doi.org/ 10.1007/s00280-011-1761-2

5. Peters GJ, Noordhuis P, Van Groeningen CJ, Giaccone G, Holwerda U, Voorn D, Schrijvers A, Schornagel JH, Beijnen JH, Fumoleau P, Schellens JH (2004) The effect of food on the pharmacokinetics of S-1 after single oral administration to patients with solid tumors. Clin Cancer Res 10: 4072–4076

**Supplementary Figures**

**Supplementary Fig. 1** Mechanism of action of TAS-114 when used in combination with 5-FU drugs

*5-FU*, 5-fluorouracil; *DPD*, dihydropyrimidine dehydrogenase; *dUTPase*, deoxyuridine triphosphatase; *TS*, thymidylate synthase


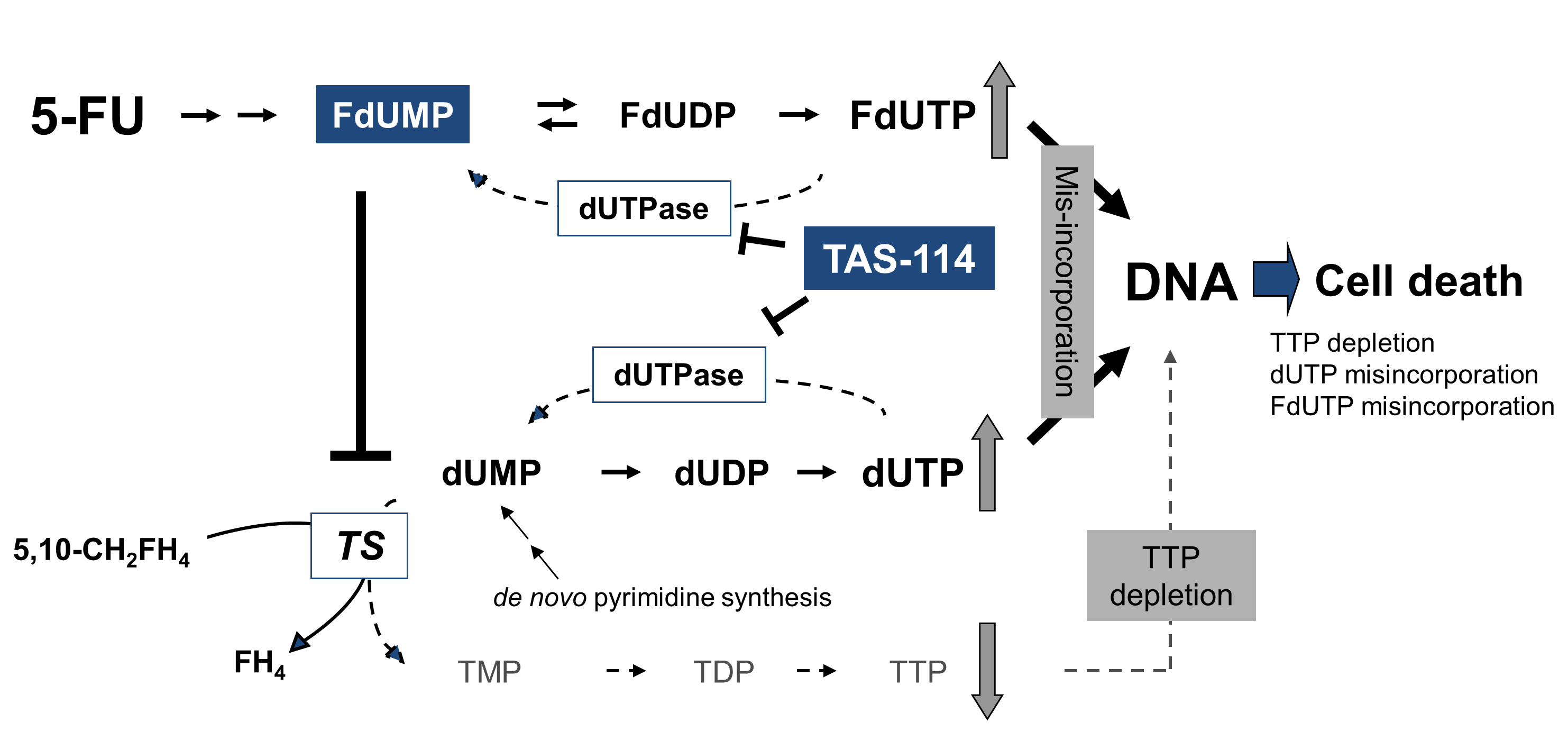


**Supplementary Fig. 2** Correlation of AUC_0-last_ on C1D1 between TAS-114 and 5-FU (a), FT (b), CDHP (c), and Oxo (d)

The p-value was calculated by linear regression analysis.

*5-FU*, 5-fluorouracil; *AUC_0-last_*, area under plasma concentration-time curve from time 0 to last quantifiable concentration; *C1D1*, Day 1 of Cycle 1; *FT*, tegafur; *CDHP*, 5-chloro-2,4-dihydroxypyridine; *Oxo,* potassium oxonate

(a)


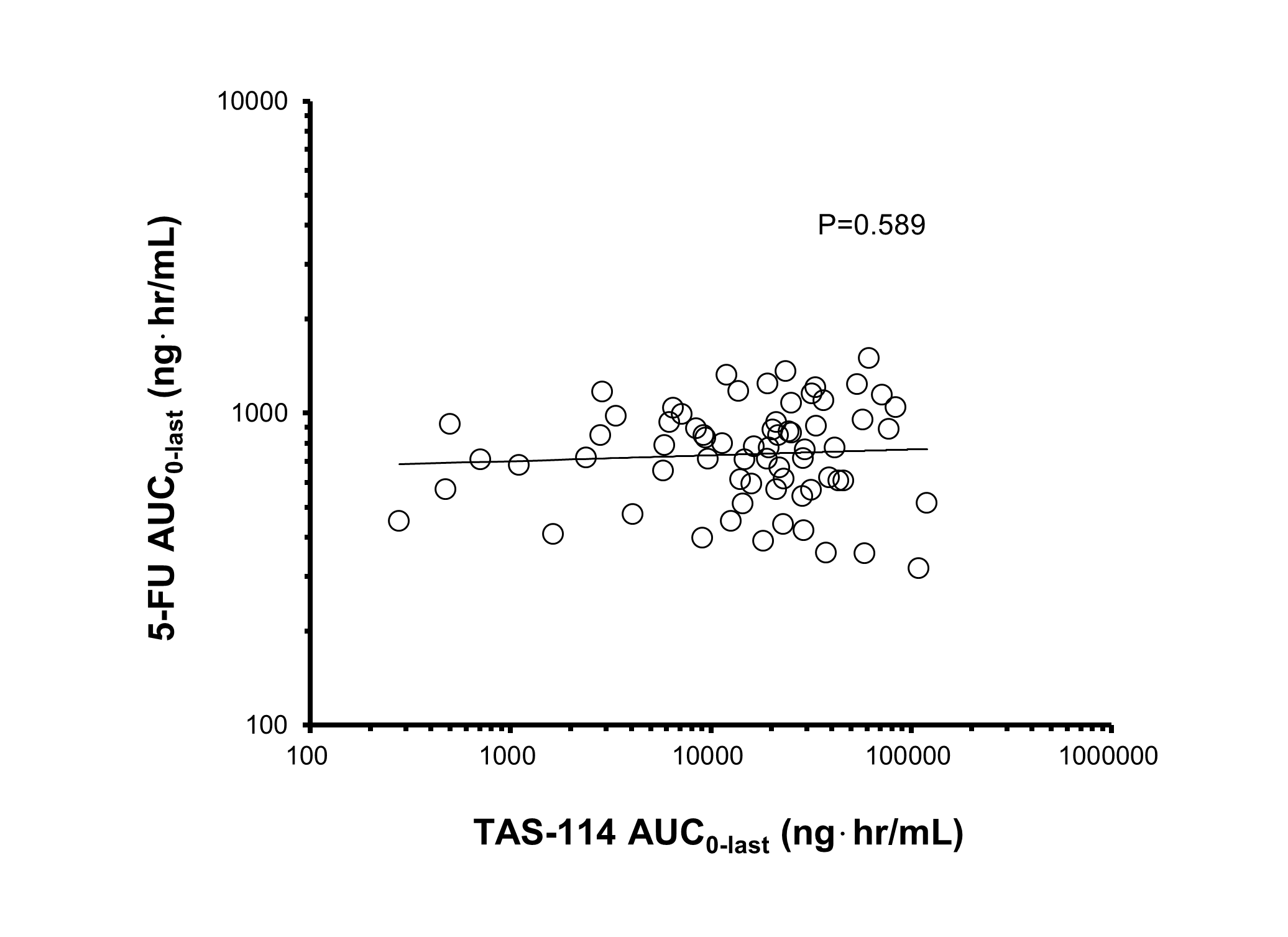


(b)


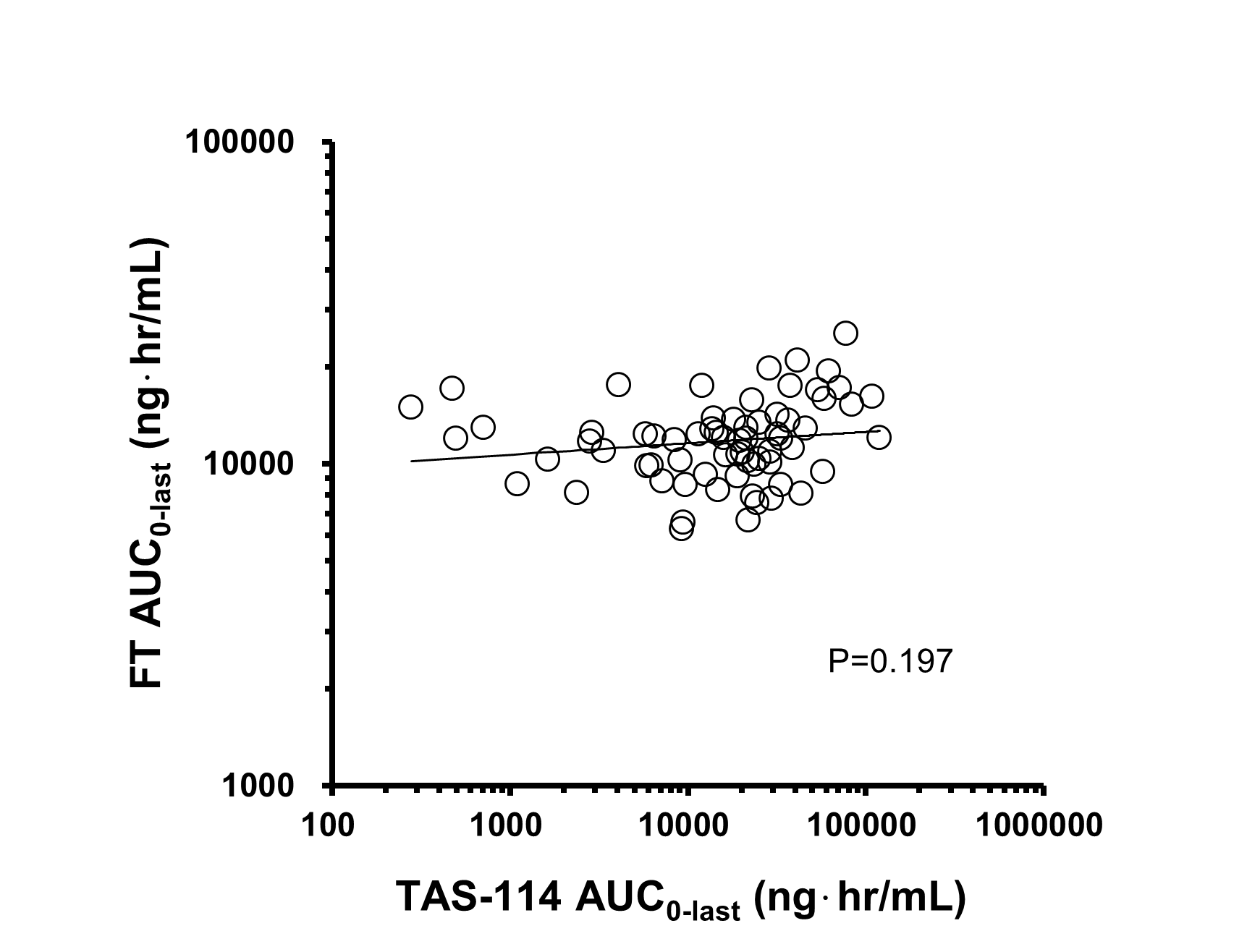


(c)


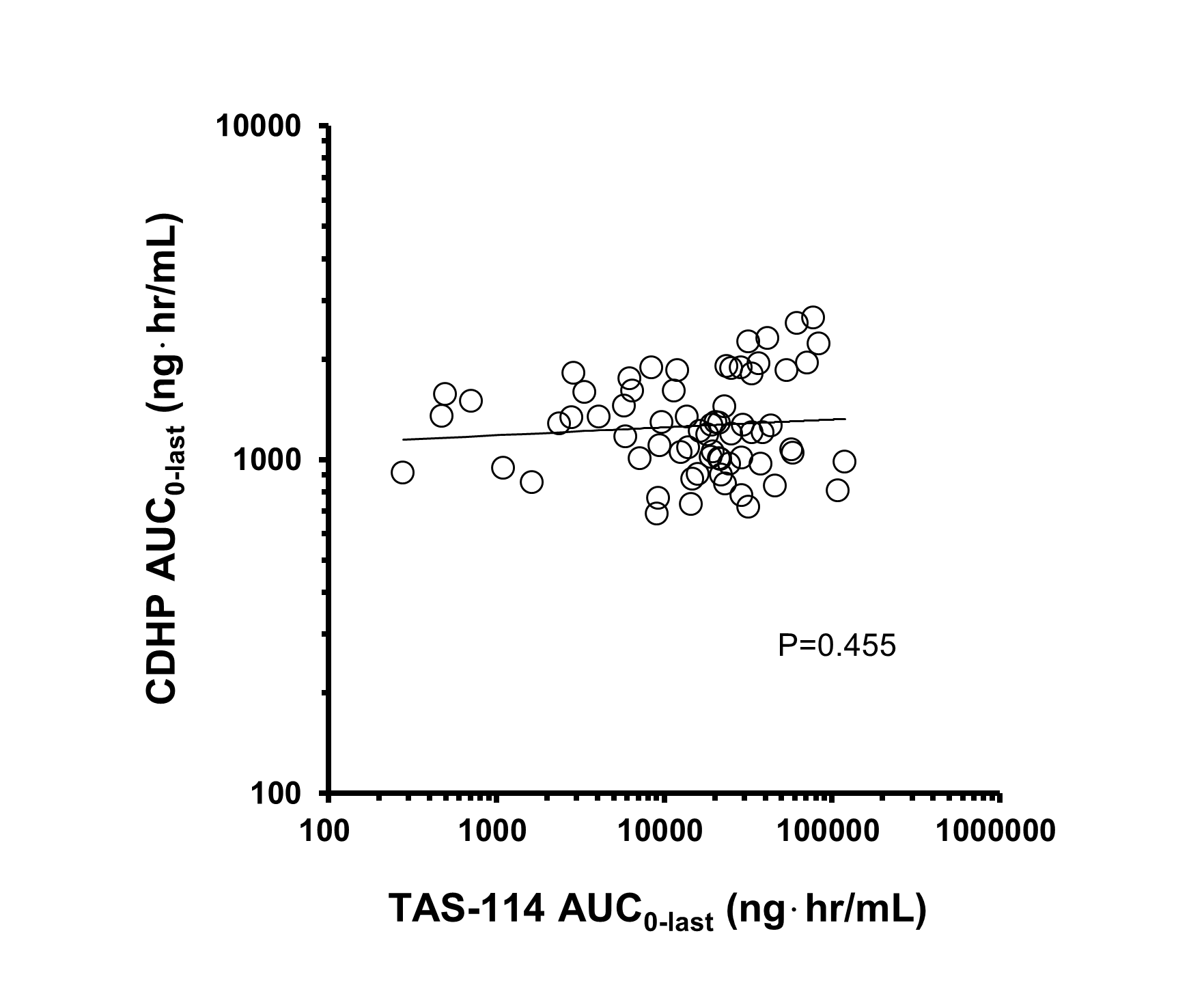


(d)


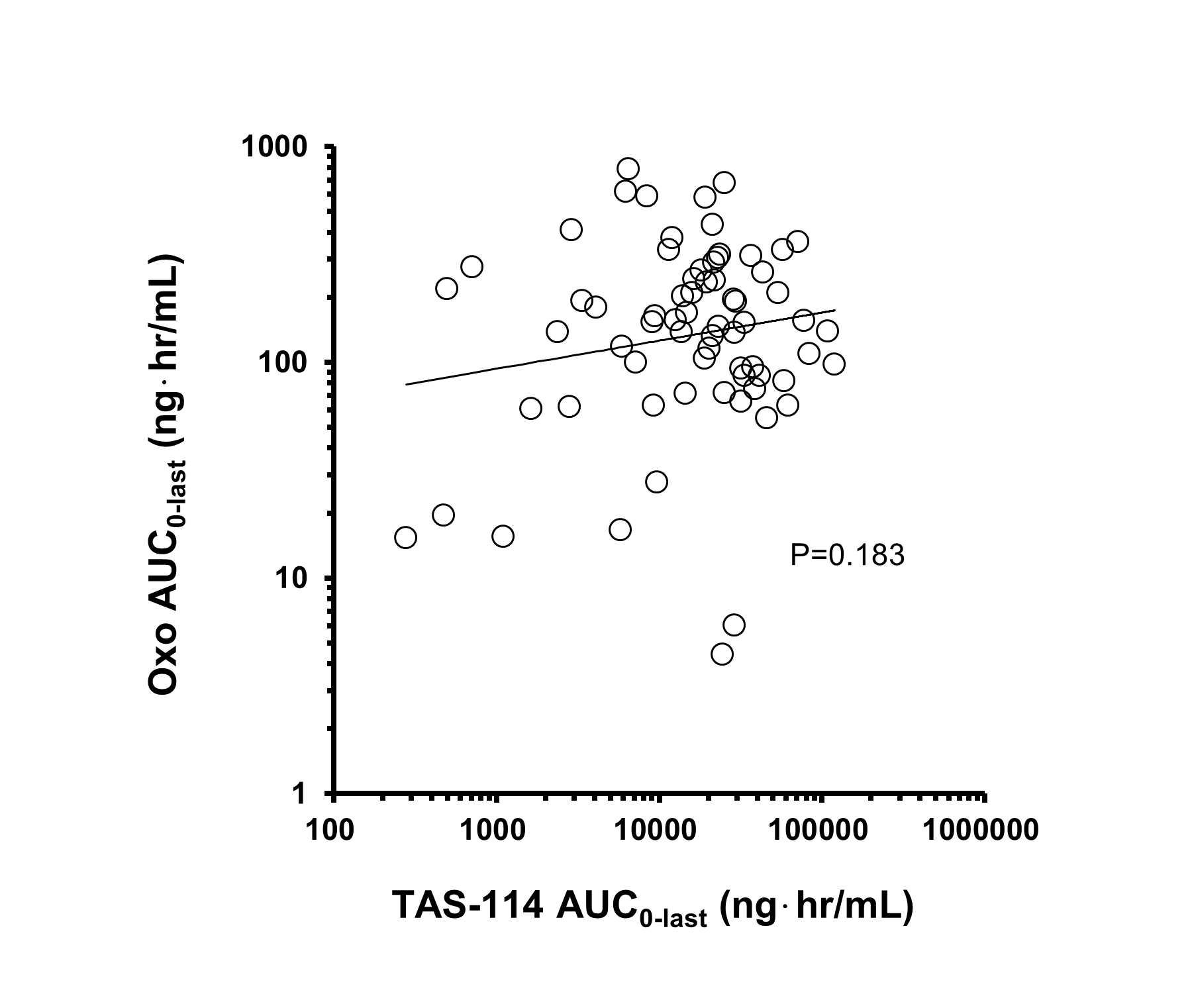


**Supplementary Fig. 3** Comparison of C_max_ (a) and AUC_0-last_ (b) of 5-FU after administration of S-1 between 30 mg/m^2^ in fasting condition and 36 mg/m^2^ in fed condition

*5-FU*, 5-fluorouracil; *C_max_*, maximum concentration; *AUC_0-last_*, area under plasma concentration-time curve from time 0 to last quantifiable concentration; *NS*, not significant

(a)


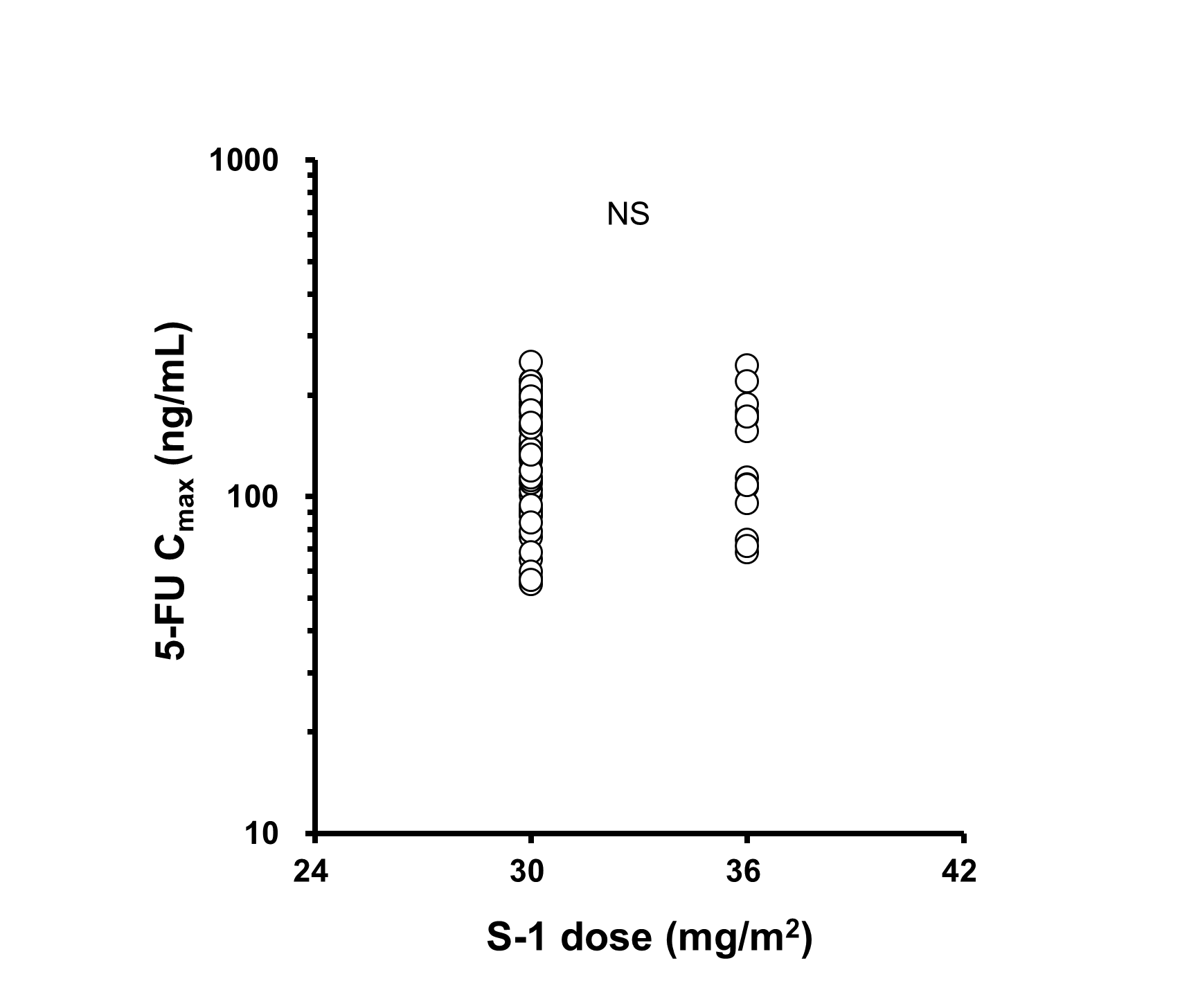


(b)


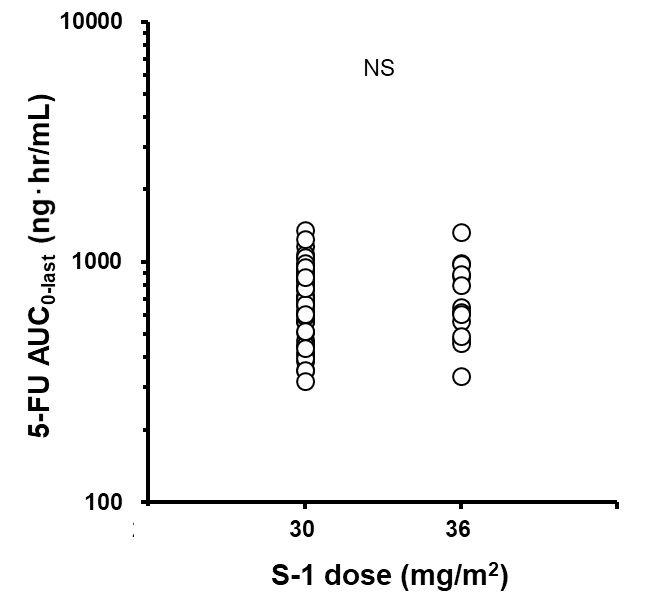


**Supplementary Fig. 4** Comparison of the ratio of urine 6-OHF concentration to urine cortisol concentration (6-OHF/F) on Day 0 with that on Days 1, 7, 14, and 21 in Cycle 1 at a TAS-114 dose of 120 (a), 160 (b), 200 (c), and 240 (d) mg/m^2^

*6OHF*, 6 beta-hydroxycortisol; *6-OHF/F*, ratio of urine 6-OHF concentration to urine cortisol concentration; *NS*, not significant

*: p < 0.05, **: p < 0.01, ***: p < 0.001

(a)


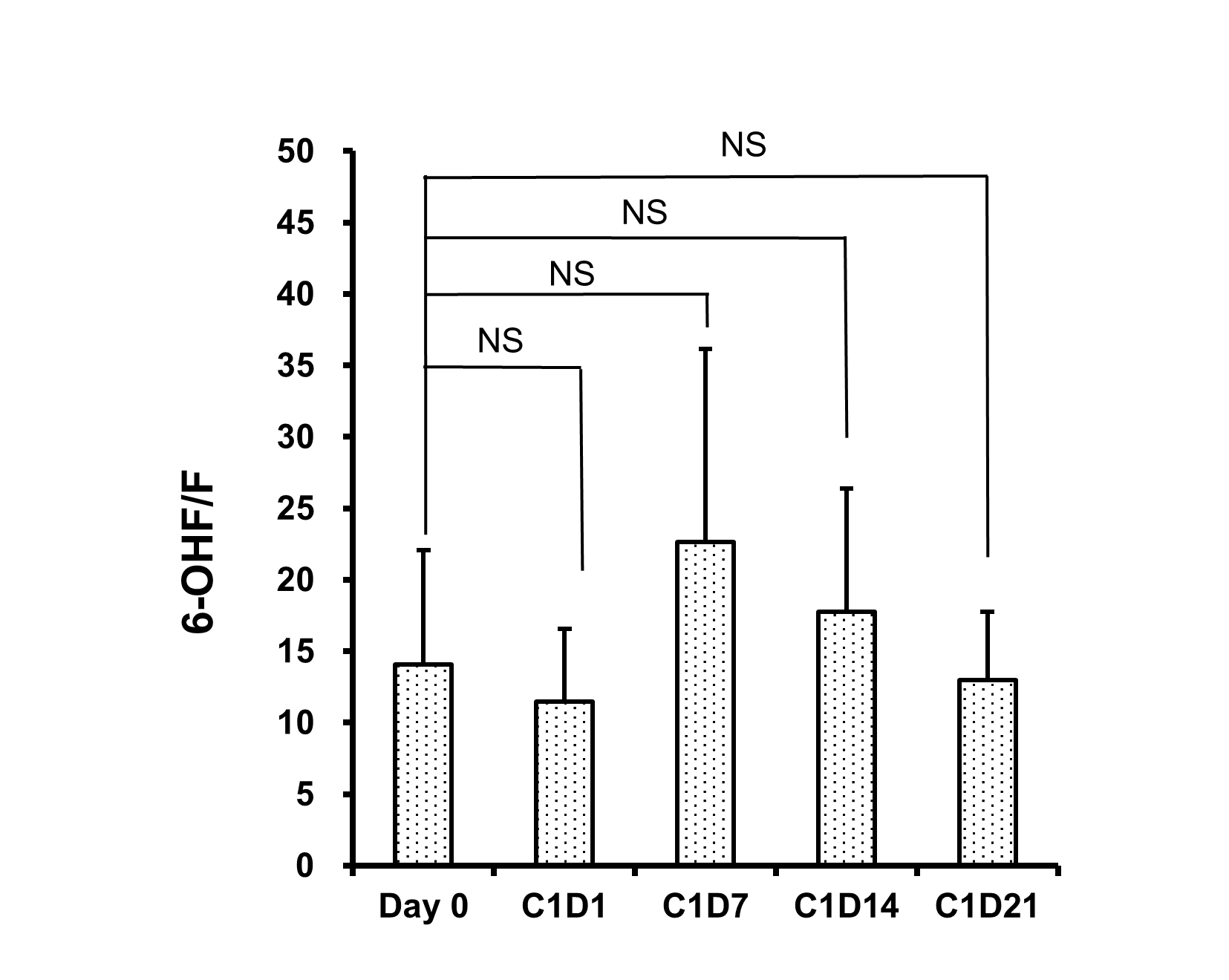


(b)


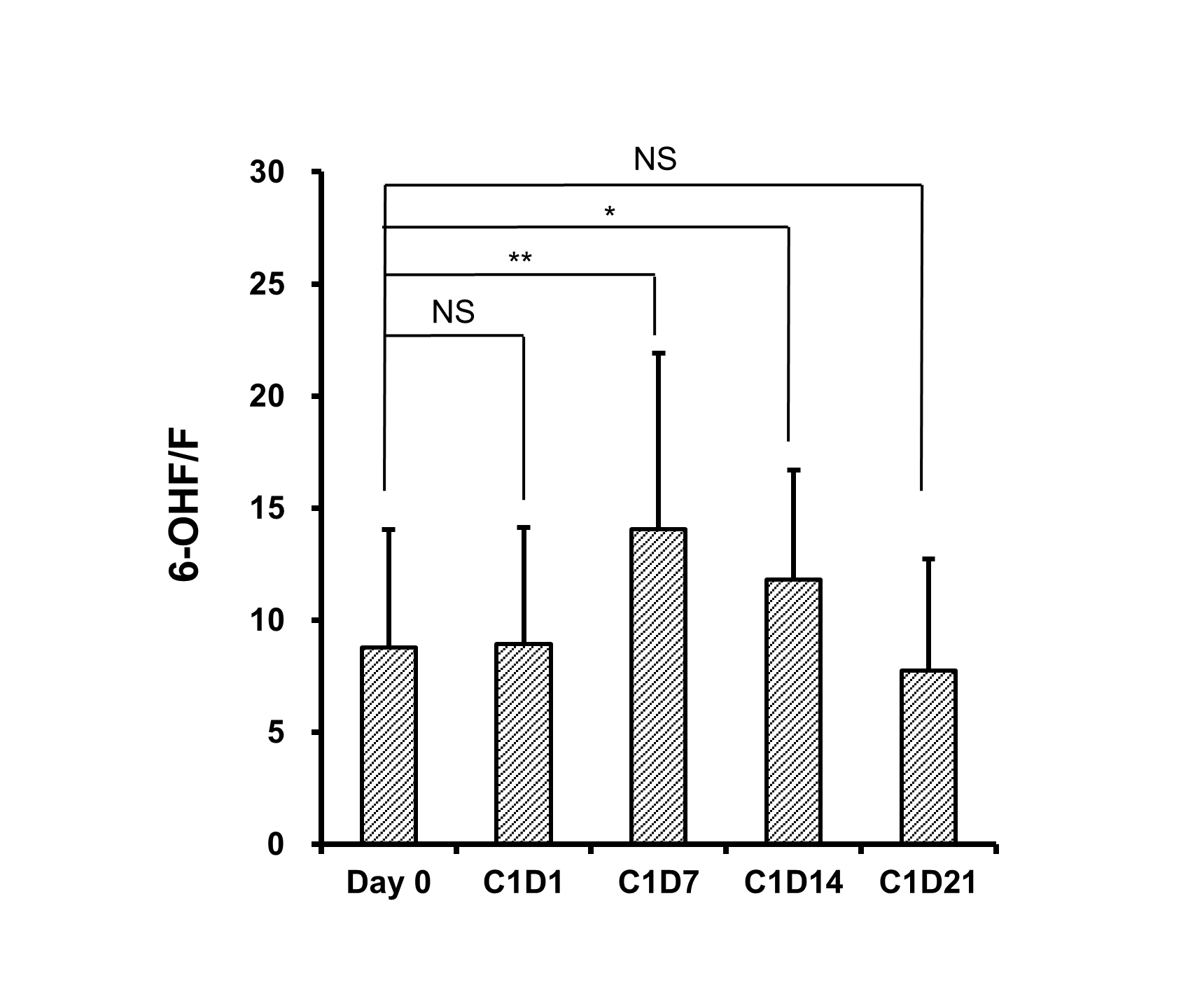


(c)


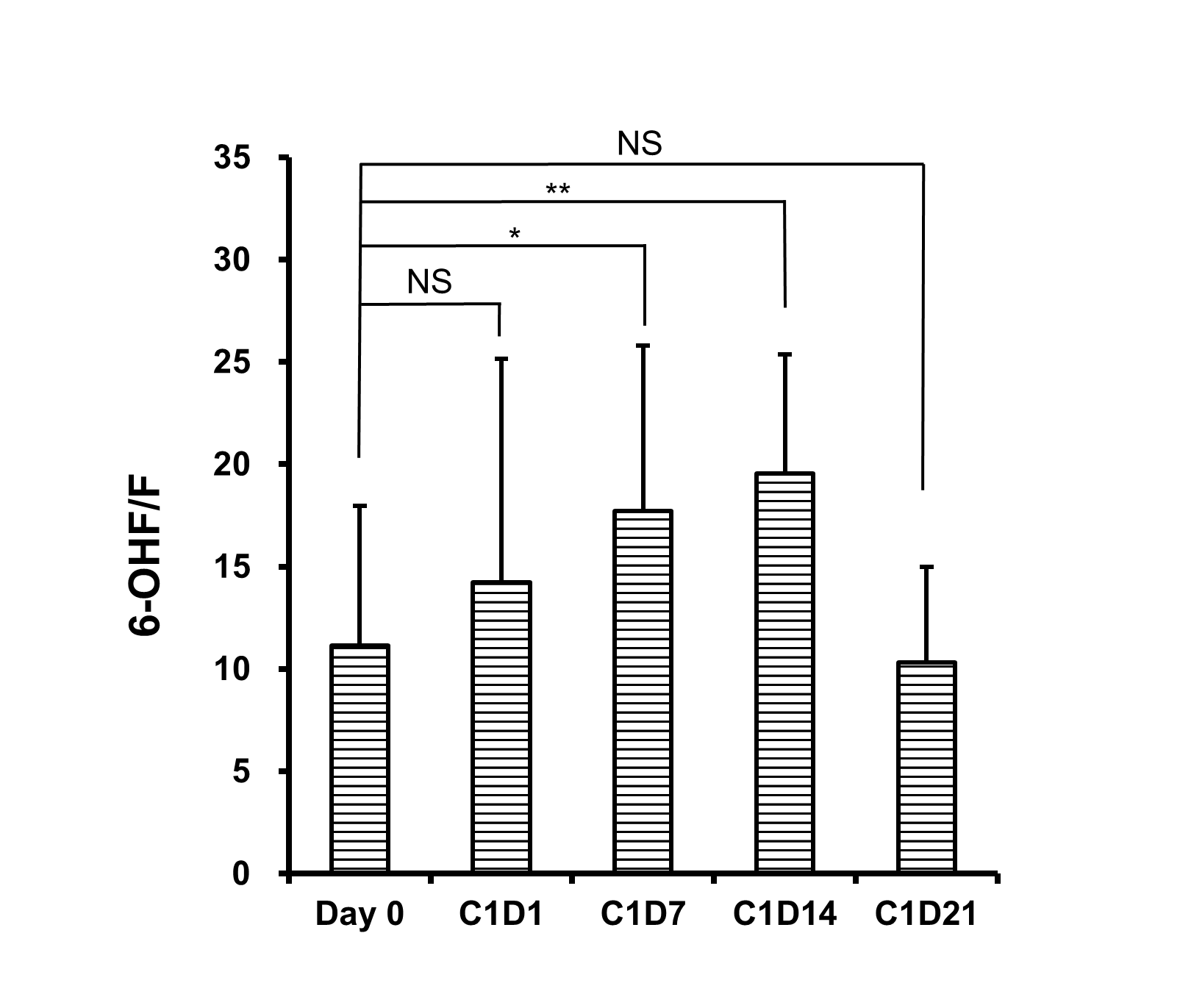


(d)


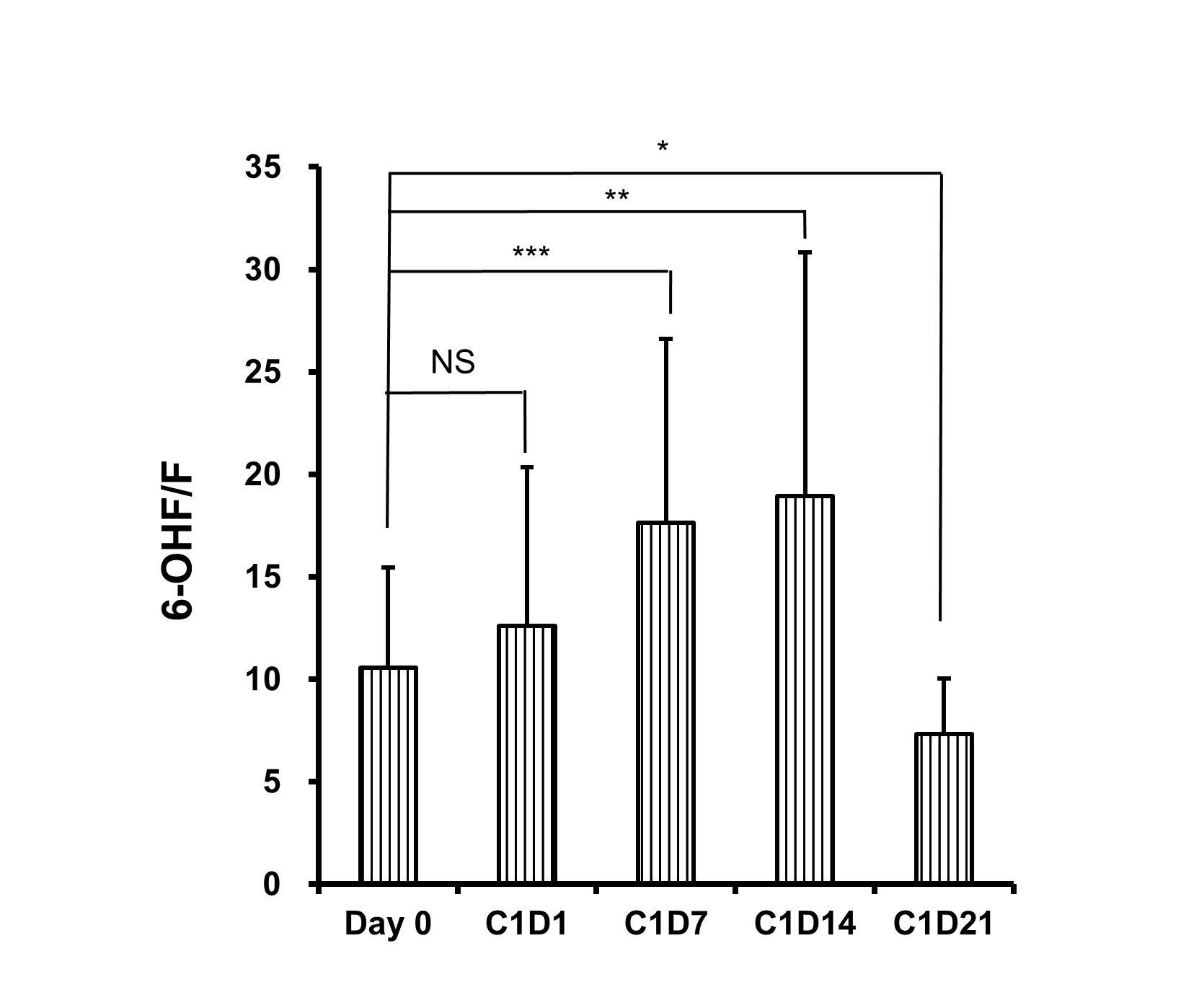


**Supplementary Tables**

**Supplementary Table 1** ORR, PFS, and number of prior systemic regimens according to cancer type

| **Cancer type** | **Number of patients (responder/total)** | **ORR (%)** | **PFS (median), months** | **Median number of prior systemic regimens** |
| --- | --- | --- | --- | --- |
| Pancreas | 0/17 | 0 | 1.4 | 2.0 |
| NSCLC | 5/16 | 18.8 (31.3^a^) | 4.1 | 3.5 |
| Colorectal | 0/10 | 0 | 1.5 | 3.0 |
| Bladder | 0/6 | 0 | 2.5 | 1.0 |
| p-NET | 2/4 | 50 | 14 | 3.0 |
| Gastric | 2/3 | 33.3 (66.7^a^) | 2.8 | 3.0 |
| Other | 1/18 | 5.6 | 3.1 | 3.0 |

*Abbreviations: NSCLC,* non-small cell lung cancer; *ORR,* overall response rate; *PFS,* progression-free survival; *p-NET,* pancreatic neuroendocrine tumor

^a^ Including unconfirmed partial response

**Supplementary Table 2A** Pharmacokinetic parameters of TAS-114

| **S-1**  **(mg/m^2^)** | **TAS-114**  **(mg/m^2^)** |  | **Day 1 in Cycle 1** | | | | | **Day 7 in Cycle 1** | | | | | **Day 14 in Cycle 1** | | | | | **Day 1 in Cycle 2** | | | | |
| --- | --- | --- | --- | --- | --- | --- | --- | --- | --- | --- | --- | --- | --- | --- | --- | --- | --- | --- | --- | --- | --- | --- |
|  |  |  | **n** | **C_max_** | **t_max_** | **AUC_0-last_** | **t_1/2_** | **n** | **C_max, Day 7_** | **t_max, Day 7_** | **AUC_0-12, Day 7_** | **t_1/2, Day 7_** | **n** | **C_max, Day 14_** | **t_max, Day 14_** | **AUC_0-12, Day 14_** | **t_1/2, Day 14_** | **n** | **C_max, Day 1 (Cycle 2)_** | **t_max, Day 1 (Cycle 2)_** | **AUC_0-12, Day 1 (Cycle 2)_** | **t_1/2, Day 1 (Cycle 2)_** |
|  |  |  |  | **(ng/mL)** | **(hr)** | **(ng·hr/mL)** | **(hr)** |  | **(ng/mL)** | **(hr)** | **(ng·hr/mL)** | **(hr)** |  | **(ng/mL)** | **(hr)** | **(ng·hr/mL)** | **(hr)** |  | **(ng/mL)** | **(hr)** | **(ng·hr/mL)** | **(hr)** |
| 30 | 5 | Mean | 3 | 125 | 1.3 | 418 | 2.1 | - | - | - | - | - | - | - | - | - | - | - | - | - | - | - |
|  |  | SD |  | 62 | 0.6 | 122 | 0.3 |  | - | - | - | - |  | - | - | - | - |  | - | - | - | - |
|  | 10 | Mean | 6 | 569 | 2 | 2213 | 2.6 | - | - | - | - | - | - | - | - | - | - | - | - | - | - | - |
|  |  | SD |  | 325 | 1.5 | 1316 | 0.5 |  | - | - | - | - |  | - | - | - | - |  | - | - | - | - |
|  | 20 | Mean | 3 | 793 | 2.3 | 3835 | 2.9 | - | - | - | - | - | - | - | - | - | - | - | - | - | - | - |
|  |  | SD |  | 122 | 1.5 | 1703 | 1.5 |  | - | - | - | - |  | - | - | - | - |  | - | - | - | - |
|  | 40 | Mean | 3 | 1777 | 1 | 6202 | 2.2 | - | - | - | - | - | - | - | - | - | - | - | - | - | - | - |
|  |  | SD |  | 290 | 0 | 294 | 0.3 |  | - | - | - | - |  | - | - | - | - |  | - | - | - | - |
|  | 60 | Mean | 3 | 2085 | 4.7 | 12263 | 3 | - | - | - | - | - | - | - | - | - | - | - | - | - | - | - |
|  |  | SD |  | 932 | 1.2 | 2432 | NC |  | - | - | - | - |  | - | - | - | - |  | - | - | - | - |
|  | 90 | Mean | 3 | 3567 | 1.7 | 16272 | 2.3 | - | - | - | - | - | - | - | - | - | - | - | - | - | - | - |
|  |  | SD |  | 1054 | 0.6 | 5956 | 0.3 |  | - | - | - | - |  | - | - | - | - |  | - | - | - | - |
|  | 120 | Mean | 10 | 3324 | 3.5 | 16430 | 2.5 | 6 | 2949 | 2.5 | 15108 | 2.2 | 6 | 2648 | 3.7 | 16906 | 2.8 | 5 | 3306 | 3.2 | 20476 | 5 |
|  |  | SD |  | 1738 | 3.4 | 6010 | 0.4 |  | 230 | 1.2 | 2674 | 0.2 |  | 1190 | 0.8 | 9010 | 0.8 |  | 432 | 1.1 | 4691 | 4 |
|  | 160 | Mean | 12 | 5459 | 2.3 | 32144 | 2.7 | 11 | 5306 | 2.3 | 26600 | 2.3 | 10 | 4484 | 2.4 | 24194 | 2.2 | 9 | 6262 | 3.4 | 44184 | 4.2^b^ |
|  |  | SD |  | 2846 | 1.4 | 27198 | 0.9 |  | 3315 | 1.2 | 24380 | 0.7 |  | 3134 | 1.2 | 18882 | 0.6 |  | 4287 | 2.1 | 41836 | 3.2^b^ |
|  | 200 | Mean | 3 | 6787 | 2 | 35233 | 3.4 | 3 | 5172 | 2.7 | 22507 | 2.4 | 3 | 4041 | 2.3 | 19247 | 2.4 | 3 | 6284 | 3 | 40419 | 3.6 |
|  |  | SD |  | 1716 | 1.7 | 5354 | 0.8 |  | 523 | 1.2 | 962 | 0.6 |  | 1327 | 1.5 | 1329 | 0.5 |  | 853 | 1.7 | 6956 | 1.2 |
|  | 240 | Mean | 10 | 8100 | 2.2 | 44177 | 2.6 | 9 | 6453 | 1.8 | 28878 | 2.3 | 9 | 6677 | 1.9 | 31623 | 2.3 | 9 | 7483 | 2.7 | 46769 | 3 |
|  |  | SD |  | 4327 | 1 | 34353 | 1.1 |  | 2606 | 1 | 15107 | 0.5 |  | 2687 | 0.3 | 20215 | 0.7 |  | 2457 | 1.3 | 24654 | 1.2 |
| 36 | 200 | Mean | 7 | 6975 | 2 | 35275 | 2.8 | 6 | 5583 | 2 | 26327 | 2.2 | 6 | 4510 | 3.2 | 25136 | 2.5^a^ | 6 | 5601 | 3.3 | 37723 | 3.2^c^ |
|  |  | SD |  | 3878 | 1 | 22230 | 1.3 |  | 2858 | 1.1 | 13989 | 0.5 |  | 1119 | 1.8 | 12452 | 1^a^ |  | 2045 | 1.6 | 21198 | 2.1^c^ |
|  | 240 | Mean | 5 | 9438 | 2 | 52484 | 3 | 4 | 7308 | 1.8 | 32843 | 2.3 | 4 | 6845 | 1.8 | 36596 | 2.3 | 4 | 10413 | 2.3 | 67715 | 4.1 |
|  |  | SD |  | 2484 | 0 | 15374 | 0.8 |  | 1548 | 0.5 | 6716 | 0.4 |  | 2294 | 0.5 | 10041 | 0.2 |  | 4623 | 1.3 | 29673 | 0.9 |

*Abbreviations: AUC_0-12_,* area under plasma concentration-time curve from time 0 to 12 hours; *AUC_0-last_,* area under plasma concentration-time curve from time 0 to last quantifiable concentration; *C_max_,* maximum plasma concentration; *NC,* not calculable; *SD,* standard deviation; *t_max_,* the time after administration of a drug when the maximum plasma concentration is reached; *t_1/2_,* time required for the concentration to fall to 50% of its current value

^a^ n = 5; b n = 7; c n = 5

**Supplementary Table 2B** Pharmacokinetic parameters of S-1 components

| **S-1**  **(mg/m^2^)** | **TAS-114**  **(mg/m^2^)** |  | **5-FU** | | | | | **FT** | | | | | **CDHP** | | | | | **Oxo** | | | | |
| --- | --- | --- | --- | --- | --- | --- | --- | --- | --- | --- | --- | --- | --- | --- | --- | --- | --- | --- | --- | --- | --- | --- |
|  |  |  | **n** | **C_max_** | **t_max_** | **AUC_0-last_** | **t_1/2_**^a^ | **n** | **C_max_** | **t_max_** | **AUC_0-last_** | **t_1/2_** | **n** | **C_max_** | **t_max_** | **AUC_0-last_** | **t_1/2_**^b^ | **n** | **C_max_** | **t_max_** | **AUC_0-last_** | **t_1/2_**^c^ |
|  |  |  |  | **(ng/mL)** | **(hr)** | **(ng·hr/mL)** | **(hr)** |  | **(ng/mL)** | **(hr)** | **(ng·hr/mL)** | **(hr)** |  | **(ng/mL)** | **(hr)** | **(ng·hr/mL)** | **(hr)** |  | **(ng/mL)** | **(hr)** | **(ng·hr/mL)** | **(hr)** |
| 30 | 5 | Mean | 3 | 127.6 | 3.3 | 647.2 | 1.8 | 3 | 1926 | 1.7 | 14640 | 8.3 | 3 | 334.1 | 1.7 | 1274.8 | 2.8 | 3 | 26.2 | 1.7 | 84.4 | 1.6 |
|  |  | SD |  | 54.7 | 1.2 | 245.2 | 0.1 |  | 167 | 0.6 | 2552 | 0.4 |  | 138.6 | 0.6 | 333.7 | 0.2 |  | 26.9 | 0.6 | 116.1 | NC |
|  | 10 | Mean | 6 | 139.1 | 2.5 | 661.7 | 1.6 | 6 | 1760 | 1.7 | 11393 | 7.9 | 6 | 322.7 | 1.8 | 1250.8 | 2.6 | 6 | 37.5 | 2.3 | 143.3 | 1.6 |
|  |  | SD |  | 54 | 1.2 | 203.3 | 0.6 |  | 410 | 1.2 | 3461 | 2 |  | 118.8 | 1.2 | 296.6 | 0.5 |  | 24.7 | 1.4 | 93.9 | 0.1 |
|  | 20 | Mean | 3 | 155.1 | 3.3 | 889 | 2 | 3 | 1750 | 2.7 | 12148 | 8.4 | 3 | 302.3 | 2.7 | 1531.8 | 3.3 | 3 | 41.5 | 2 | 163 | 2 |
|  |  | SD |  | 47.2 | 1.2 | 259.1 | 0.4 |  | 285 | 1.2 | 386 | 2 |  | 52.2 | 1.2 | 246.4 | 0.6 |  | 50.4 | 0 | 215.3 | NC |
|  | 40 | Mean | 3 | 191.4 | 2 | 919.9 | 1.6 | 3 | 1820 | 1 | 10608 | 6.8 | 3 | 367 | 1.7 | 1506.3 | 2.7 | 3 | 142 | 1.7 | 506.7 | 2 |
|  |  | SD |  | 7.8 | 0 | 125.6 | 0.1 |  | 295 | 0 | 1337 | 0.5 |  | 53.4 | 0.6 | 298.7 | 0.2 |  | 105.5 | 0.6 | 347.1 | 0.5 |
|  | 60 | Mean | 3 | 96.4 | 4 | 558.2 | 2 | 3 | 1243 | 3.3 | 10073 | 7.5 | 3 | 165.8 | 3.3 | 1024.4 | 3.3 | 3 | 19 | 2 | 85.3 | 5.3 |
|  |  | SD |  | 12.7 | 0 | 137.2 | 0.3 |  | 289 | 1.2 | 2094 | 0.9 |  | 38.6 | 1.2 | 279 | 0.5 |  | 8.7 | 0 | 65.8 | NC |
|  | 90 | Mean | 3 | 146.7 | 3.3 | 810.2 | 1.9 | 3 | 1425 | 1.7 | 8825 | 6.8 | 3 | 214.7 | 2.3 | 1135.8 | 3.6 | 3 | 24.1 | 3.3 | 127.7 | 2.4 |
|  |  | SD |  | 10.3 | 1.2 | 88.9 | 0.6 |  | 368 | 0.6 | 2139 | 1.3 |  | 63.9 | 1.5 | 142.4 | 1.5 |  | 2.5 | 2.3 | 31.3 | NC |
|  | 120 | Mean | 10 | 126.3 | 4 | 745.1 | 1.9 | 10 | 1464 | 2.9 | 11969 | 8.5 | 10 | 234.6 | 2.8 | 1373.5 | 3.3 | 10 | 58.7 | 3.2 | 324.9 | 3.3 |
|  |  | SD |  | 51.4 | 0.9 | 311.9 | 0.3 |  | 249 | 1.5 | 2245 | 2 |  | 85.9 | 1.4 | 436.7 | 0.3 |  | 33.4 | 2.3 | 173.9 | 0.9 |
|  | 160 | Mean | 12 | 141.5 | 3.2 | 715.6 | 1.7 | 12 | 1469 | 2.3 | 10694 | 7.6 | 12 | 207.7 | 2.3 | 978.1 | 2.9 | 12 | 32.4 | 2.1 | 155.2 | 3.6 |
|  |  | SD |  | 51.4 | 1 | 217.4 | 0.4 |  | 307 | 1.1 | 3108 | 2.3 |  | 47.2 | 0.8 | 106 | 0.6 |  | 13 | 0.7 | 77.8 | 1 |
|  | 200 | Mean | 3 | 81.7 | 3.3 | 465.7 | 2.3 | 3 | 1644 | 2.7 | 13151 | 10.3 | 3 | 229.8 | 2.7 | 984.7 | 3.2 | 3 | 17.5 | 2.7 | 58.7 | NC |
|  |  | SD |  | 26.2 | 1.2 | 139 | 0.6 |  | 349 | 1.2 | 3723 | 2.3 |  | 69 | 1.2 | 211.8 | 0.5 |  | 10.2 | 1.2 | 46.7 | NC |
|  | 240 | Mean | 10 | 153.7 | 3.4 | 796.1 | 1.8 | 10 | 1463 | 2.4 | 10404 | 7 | 10 | 230.9 | 2.7 | 1202.6 | 3.3 | 10 | 45.2 | 3 | 219.9 | 8.2 |
|  |  | SD |  | 51.2 | 1 | 238.1 | 0.6 |  | 296 | 1.2 | 2634 | 2 |  | 72.5 | 1.2 | 415.1 | 0.5 |  | 39.9 | 2.3 | 178.2 | 10.9 |
| 36 | 200 | Mean | 7 | 162.3 | 4.3 | 969.2 | 2.3 | 7 | 2332 | 2.1 | 16542 | 9.9 | 7 | 333.5 | 3.7 | 1819.5 | 4.6 | 7 | 31.4 | 3.3 | 189 | 8.6 |
|  |  | SD |  | 49.8 | 3.5 | 300.3 | 0.6 |  | 646 | 1.3 | 4645 | 3.5 |  | 129.9 | 3.7 | 593.7 | 1.9 |  | 14.4 | 3.9 | 115.9 | 8.6 |
|  | 240 | Mean | 5 | 176.7 | 3.6 | 1170.7 | 2.5 | 5 | 2321 | 1.4 | 17250 | 8.6 | 5 | 417.5 | 2 | 2091.9 | 3.5 | 5 | 32.2 | 2.2 | 161 | 2.9 |
|  |  | SD |  | 44.8 | 0.9 | 259.4 | 0.9 |  | 358 | 0.5 | 3391 | 1.6 |  | 66.3 | 0 | 330.1 | 0.7 |  | 17.9 | 1.1 | 125.3 | NC |

*Abbreviations: 5-FU,* 5-fluorouracil; *AUC_0-last_,* area under plasma concentration-time curve from time 0 to last quantifiable concentration; *CDHP,* 5-chloro-2,4-dihydroxypyridine; *C_max_,* maximum plasma concentration; *FT,* tegafur; *NC,* not calculable; *Oxo,* potassium oxonate; *SD,* standard deviation; *t_1/2_,* time required for the concentration to fall to 50% of its current value; *t_max_,* the time after administration of a drug when the maximum plasma concentration is reached

^a^ S-1 30 mg/m^2^ + TAS-114 160 mg/m^2^, n=11; S-1 36 mg/m^2^ + TAS-114 200 mg/m^2^, n=6

^b^ S-1 30 mg/m^2^ + TAS-114 120 mg/m^2^, n=9; S-1 36 mg/m^2^ + TAS-114 200 mg/m^2^, n=6

^c^ S-1 30 mg/m^2^ + TAS-114 5 mg/m^2^, n=1; S-1 30 mg/m^2^ + TAS-114 10 mg/m^2^, n=3; S-1 30 mg/m^2^ + TAS-114 20 mg/m^2^, n=1; S-1 30 mg/m^2^ + TAS-114 40 mg/m^2^, n=2;

S-1 30 mg/m^2^ + TAS-114 60 mg/m^2^, n=1; S-1 30 mg/m^2^ + TAS-114 90 mg/m^2^, n=2; S-1 30 mg/m^2^ + TAS-114 120 mg/m^2^, n=8; S-1 30 mg/m^2^ + TAS-114 160 mg/m^2^, n=6;

S-1 30 mg/m^2^ + TAS-114 200 mg/m^2^, n=0; S-1 30 mg/m^2^ + TAS-114 240 mg/m^2^, n=5; S-1 36 mg/m^2^ + TAS-114 200 mg/m^2^, n=4; S-1 36 mg/m^2^ + TAS-114 240 mg/m^2^, n=2

**Supplementary Table 3** Relationships between the presence or absence of disease control and the mRNA or protein expression levels of dUTPase, DPD, TS, TP, UNG, APEX1, POLB, BRCA1, and BRCA2

|  | **CR+PR+SD (N=15)** | **PD (N=31)** | **P value**^a^ |
| --- | --- | --- | --- |
| dUTPase mRNA | | | |
| N | 14 | 23 | 0.562 |
| Mean (standard deviation) | 0.146000 (0.217289) | 0.106188 (0.129647) |
| Median (min–max) | 0.076100 (0.02280–0.87000) | 0.069400 (0.00433–0.63400) |
| DPD mRNA | | | |
| N | 14 | 22 | 0.795 |
| Mean (standard deviation) | 0.029669 (0.022841) | 0.031517 (0.034056) |
| Median (min–max) | 0.022250 (0.00345–0.07970 | 0.018200 (0.00340–0.13500) |
| TS mRNA | | | |
| N | 14 | 23 | 0.695 |
| Mean (standard deviation) | 0.028793 (0.028653) | 0.039364 (0.048083) |
| Median (min–max) | 0.019000 (0.00181–0.10200) | 0.018800 (0.00206–0.22300) |
| TP mRNA | | | |
| N | 14 | 23 | 0.481 |
| Mean (standard deviation) | 0.21209 (0.30792) | 0.15913 (0.22012) |
| Median (min–max) | 0.11300 (0.0249–1.2300 | 0.09240 (0.0135–1.0800 |
| UNG mRNA | | | |
| N | 12 | 22 | 0.928 |
| Mean (standard deviation) | 0.026158 (0.022445) | 0.025880 (0.018177) |
| Median (min–max) | 0.020000 (0.00122–0.06770) | 0.018450 (0.00308–0.05850) |
| APEX1 mRNA | | | |
| N | 14 | 23 | 0.814 |
| Mean (standard deviation) | 0.029107 (0.013973) | 0.031771 (0.018270) |
| Median (min–max) | 0.025900 (0.01140–0.06520) | 0.031800 (0.00783–0.07520) |
| POLB mRNA | | | |
| N | 12 | 22 | 0.272 |
| Mean (standard deviation) | 0.009882 (0.011614) | 0.013864 (0.019818) |
| Median (min–max) | 0.005475 (0.00275–0.04450) | 0.010170 (0.00225–0.09840) |
| BRCA1 mRNA | | | |
| N | 12 | 22 | 0.957 |
| Mean (standard deviation) | 0.009379 (0.008506) | 0.008375 (0.008973) |
| Median (min–max) | 0.006170 (0.00171–0.02490) | 0.005740 (0.00102–0.04240) |
| BRCA2 mRNA | | | |
| N | 13 | 22 | 0.932 |
| Mean (standard deviation) | 0.0029002 (0.0021596) | 0.0027955 (0.0020051) |
| Median (min–max) | 0.0023500 (0.000732–0.007770) | 0.0023950 (0.000205–0.006300) |
| dUTPase IHC H-score (nuclear) | | | |
| N | 15 | 31 | 0.021 |
| Mean (standard deviation) | 33.7 (22.2) | 61.5 (40.4) |
| Median (min–max) | 29.0 (0–79) | 57.0 (0–173) |
| dUTPase IHC H-score (Cytosol) | | | |
| N | 15 | 31 | 0.796 |
| Mean (standard deviation) | 148.7 (44.3) | 141.8 (51.8) |
| Median (min–max) | 121.0 (100–219) | 139.0 (0–294) |
| DPD IHC H-score | | | |
| N | 14 | 25 | 0.815 |
| Mean (standard deviation) | 172.8 (55.7) | 175.0 (72.9) |
| Median (min–max) | 189.0 (58–283) | 190.0 (5–298) |
| TS IHC H-score (cytosol) | | | |
| N | 15 | 29 | 0.359 |
| Mean (standard deviation) | 167.3 (71.1) | 173.5 (48.0) |
| Median (min–max) | 145.0 (96–299) | 189.0 (86–290) |
| TP IHC H-score (cytosol) | | | |
| N | 14 | 25 | 0.537 |
| Mean (standard deviation) | 54.0 (60.6) | 53.2 (72.8) |
| Median (min–max) | 27.0 (0–201) | 18.0 (0–239) |
| BRCA1 IHC H-score | | | |
| N | 15 | 29 | 0.004 |
| Mean (standard deviation) | 142.6 (43.9) | 189.2 (51.1) |
| Median (min–max) | 138.0 (51–217) | 195.0 (34–271) |

*Abbreviations: APEX1,* apurinic/apyrimidinic endodeoxyribonuclease 1; *BRCA1,* breast cancer 1, early onset; *BRCA2,* breast cancer 2, early onset; *CR,* complete response; *dUTPase,* deoxyuridine triphosphatase; *IHC,* immunohistochemistry; *PD,* progressive disease; *PR,* partial response; *SD,* stable disease; *POLB,* DNA polymerase beta; *TP,* thymidine phosphorylase; *TS,* thymidylate synthase; *UNG,* uracil-DNA glycosylase

Analysis set: PGx Evaluable Patients

^a^ Wilcoxon’s rank sum test

**Supplementary Table 4** Relationships between PFS and mRNA or protein expression levels of dUTPase, DPD, TS, TP, UNG, APEX1, POLB, BRCA1, and BRCA2 (Cutoff value: median)

|  | **High group** | **Low group** | **Hazard ratio** | **P value**^a^ |
| --- | --- | --- | --- | --- |
|  | **(N=19)** | **(N=18)** | **(95% CI)** |  |
| **dUTPase mRNA** | | | | |
| Number of patients (%) with an event | 18 (94.7) | 18 (100.0) |  |  |
| Censored, n (%) | 1 (5.3) | 0 (0.0) |  |  |
| Median PFS (95% CI), months | 2.8 (1.5–4.5) | 2.6 (1.4–4.1) | 0.81 (0.42–1.57) | 0.528 |
|  | **High Group** | **Low Group** |  |  |
|  | **(N=19)** | **(N=17)** |  |  |
| **DPD mRNA** | | | | |
| Number of patients (%) with an event | 18 (94.7) | 17 (100.0) |  |  |
| Censored, n (%) | 1 (5.3) | 0 (0.0) |  |  |
| Median PFS (95% CI), months | 2.8 (1.4–5.8) | 2.6 (1.4–4.4) | 0.78 (0.39–1.53) | 0.459 |
|  | **High Group** | **Low Group** |  |  |
|  | **(N=19)** | **(N=18)** |  |  |
| **TS mRNA** | | | | |
| Number of patients (%) with an event | 18 (94.7) | 18 (100.0) |  |  |
| Censored, n (%) | 1 (5.3) | 0 (0.0) |  |  |
| Median PFS (95% CI), months | 2.8 (1.5–4.4) | 2.6 (1.4–5.6) | 1.34 (0.66–2.75) | 0.412 |
|  | **High Group** | **Low Group** |  |  |
|  | **(N=19)** | **(N=18)** |  |  |
| **TP mRNA** | | | | |
| Number of patients (%) with an event | 18 (94.7) | 18 (100.0) |  |  |
| Censored, n (%) | 1 (5.3) | 0 (0.0) |  |  |
| Median PFS (95% CI), months | 2.8 (1.4–5.6) | 2.6 (2.0–3.9) | 0.91 (0.47–1.78) | 0.788 |
|  | **High Group** | **Low Group** |  |  |
|  | **(N=17)** | **(N=17)** |  |  |
| **UNG mRNA** | | | | |
| Number of patients (%) with an event | 16 (94.1) | 17 (100.0) |  |  |
| Censored, n (%) | 1 (5.9) | 0 (0.0) |  |  |
| Median PFS (95% CI), months | 3.9 (1.5–4.5) | 2.0 (1.4–4.1) | 0.68 (0.34–1.37) | 0.272 |
|  | **High Group** | **Low Group** |  |  |
|  | **(N=19)** | **(N=18)** |  |  |
| **APEX1 mRNA** | | | | |
| Number of patients (%) with an event | 19 (100.0) | 17 (94.4) |  |  |
| Censored, n (%) | 0 (0.0) | 1 (5.6) |  |  |
| Median PFS (95% CI), months | 2.6 (1.2–4.5) | 2.8 (2.0–5.6) | 1.03 (0.53–2.00) | 0.941 |
|  | **High Group** | **Low Group** |  |  |
|  | **(N=17)** | **(N=17)** |  |  |
| **POLB mRNA** | | | | |
| Number of patients (%) with an event | 16 (94.1) | 17 (100.0) |  |  |
| Censored, n (%) | 1 (5.9) | 0 (0.0) |  |  |
| Median PFS (95% CI), months | 2.5 (1.0–2.8) | 3.9 (1.4–5.6) | 1.57 (0.77–3.20) | 0.204 |
|  | **High Group** | **Low Group** |  |  |
|  | **(N=17)** | **(N=17)** |  |  |
| **BRCA1 mRNA** | | | | |
| Number of patients (%) with an event | 16 (94.1) | 17 (100.0) |  |  |
| Censored, n (%) | 1 (5.9) | 0 (0.0) |  |  |
| Median PFS (95% CI), months | 2.8 (1.5–4.5) | 2.6 (1.4–5.6) | 1.07 (0.53–2.16) | 0.854 |
|  | **High Group** | **Low Group** |  |  |
|  | **(N=18)** | **(N=17)** |  |  |
| **BRCA2 mRNA** | | | | |
| Number of patients (%) with an event | 17 (94.4) | 17 (100.0) |  |  |
| Censored, n (%) | 1 (5.6) | 0 (0.0) |  |  |
| Median PFS (95% CI), months | 2.8 (2.0–4.5) | 2.6 (1.4–5.6) | 1.26 (0.61–2.62) | 0.528 |
|  | **High Group** | **Low Group** |  |  |
|  | **(N=25)** | **(N=22)** |  |  |
| **dUTPase IHC H-score (nuclear)** | | | | |
| Number of patients (%) with an event | 24 (96.0) | 21 (95.5) |  |  |
| Censored, n (%) | 1 (4.0) | 1 (4.5) |  |  |
| Median PFS (95% CI), months | 2.0 (1.4–2.8) | 3.9 (1.5–5.8) | 1.92 (1.02–3.61) | 0.038 |
|  | **High Group** | **Low Group** |  |  |
|  | **(N=25)** | **(N=22)** |  |  |
| **dUTPase IHC H-score (cytosol)** | | | | |
| Number of patients (%) with an event | 25 (100.0) | 20 (90.9) |  |  |
| Censored, n (%) | 0 (0.0) | 2 (9.1) |  |  |
| Median PFS (95% CI), months | 2.5 (1.4–3.2) | 2.8 (1.5–5.6) | 1.22 (0.67–2.22) | 0.506 |
|  | **High Group** | **Low Group** |  |  |
|  | **(N=20)** | **(N=19)** |  |  |
| **DPD IHC H-score** | | | | |
| Number of patients (%) with an event | 19 (95.0) | 19 (100.0) |  |  |
| Censored | 1 (5.0) | 0 (0.0) |  |  |
| Median PFS (95% CI), months | 1.8 (1.4–5.6) | 2.8 (2.6–4.1) | 0.79 (0.40–1.58) | 0.506 |
|  | **High Group** | **Low Group** |  |  |
|  | **(N=23)** | **(N=22)** |  |  |
| **TS IHC H-score (cytosol)** | | | | |
| Number of patients (%) with an event | 21 (91.3) | 22 (100.0) |  |  |
| Censored, n (%) | 2 (8.7) | 0 (0.0) |  |  |
| Median PFS (95% CI), months | 2.5 (1.4–3.2) | 2.8 (1.5–4.5) | 1.56 (0.82–2.98) | 0.163 |
|  | **High Group** | **Low Group** |  |  |
|  | **(N=20)** | **(N=19)** |  |  |
| **TP IHC H-score (cytosol)** | | | | |
| Number of patients (%) with an event | 20 (100.0) | 18 (94.7) |  |  |
| Censored, n (%) | 0 (0.0) | 1 (5.3) |  |  |
| Median PFS (95% CI), months | 2.3 (1.4–5.8) | 2.8 (1.5–3.9) | 0.95 (0.50–1.84) | 0.889 |
|  | **High Group** | **Low Group** |  |  |
|  | **(N=23)** | **(N=21)** |  |  |
| **BRCA1 IHC H-score** | | | | |
| Number of patients (%) with an event | 22 (95.7) | 21 (100.0) |  |  |
| Censored, n (%) | 1 (4.3) | 0 (0.0) |  |  |
| Median PFS (95% CI), months | 1.5 (1.4–2.8) | 3.9 (2.5–5.6) | 1.90 (1.00–3.60) | 0.045 |

*Abbreviations: APEX1,* apurinic/apyrimidinic endodeoxyribonuclease 1; *BRCA1,* breast cancer 1, early onset; *BRCA2,* breast cancer 2, early onset; *CI,* confidence interval; *DPD,* dihydropyrimidine dehydrogenase; *dUTPase,* deoxyuridine triphosphatase; *IHC,* immunohistochemistry; *PD,* progressive disease; *PFS,* progression-free survival; *POLB,* DNA polymerase beta; *SD,* standard deviation; *TP,* thymidine phosphorylase; *TS,* thymidylate synthase; *UNG,* uracil-DNA glycosylase

Analysis set: PGx Evaluable Patients

^a^ logrank test
